# A *Drosophila* functional evaluation of candidates from human genome-wide association studies of type 2 diabetes and related metabolic traits identifies tissue-specific roles for *dHHEX*

**DOI:** 10.1186/1471-2164-14-136

**Published:** 2013-02-27

**Authors:** Jay Pendse, Prasanna V Ramachandran, Jianbo Na, Narisu Narisu, Jill L Fink, Ross L Cagan, Francis S Collins, Thomas J Baranski

**Affiliations:** 1 Department of Developmental and Regenerative Biology, Mount Sinai School of Medicine, New York, NY, USA; 2 Department of Medicine, Washington University School of Medicine, St. Louis, MO, USA; 3 Genome Technology Branch, National Human Genome Research Institute, National Institutes of Health, Bethesda, MD, USA

**Keywords:** Genome-wide association study, *Drosophila melanogaster*, Diabetes mellitus, type 2, Hyperglycemia, Dyslipidemias, Phylogeny, Reverse genetics, High-throughput screening assays, HHEX protein, Human

## Abstract

**Background:**

Genome-wide association studies (GWAS) identify regions of the genome that are associated with particular traits, but do not typically identify specific causative genetic elements. For example, while a large number of single nucleotide polymorphisms associated with type 2 diabetes (T2D) and related traits have been identified by human GWAS, only a few genes have functional evidence to support or to rule out a role in cellular metabolism or dietary interactions. Here, we use a recently developed *Drosophila* model in which high-sucrose feeding induces phenotypes similar to T2D to assess orthologs of human GWAS-identified candidate genes for risk of T2D and related traits.

**Results:**

Disrupting orthologs of certain T2D candidate genes (*HHEX*, *THADA*, *PPARG*, *KCNJ11*) led to sucrose-dependent toxicity. Tissue-specific knockdown of the *HHEX* ortholog *dHHEX* (*CG7056*) directed metabolic defects and enhanced lethality; for example, fat-body-specific loss of *dHHEX* led to increased hemolymph glucose and reduced insulin sensitivity.

**Conclusion:**

Candidate genes identified in human genetic studies of metabolic traits can be prioritized and functionally characterized using a simple *Drosophila* approach. To our knowledge, this is the first large-scale effort to study the functional interaction between GWAS-identified candidate genes and an environmental risk factor such as diet in a model organism system.

## Author summary

The search for genetic risk factors for common human diseases often relies on the use of linkage and association studies to establish correlation between genomic markers and disease risk. These studies require additional functional evaluation of candidate genes, including their possible interaction with diet and environment. The number of candidate genes is typically large and the development of appropriate genetic tools in mammalian systems is slow. By contrast, large-scale genetic screens, using widely available genetic tools, are routinely conducted in the fruit fly *Drosophila melanogaster*. In this study, we used *Drosophila* to screen candidate genes identified in human genome-wide scans as associated with risk of metabolic abnormalities such as type 2 diabetes. We show that a number of human candidate genes have fly orthologs that play an important role in *Drosophila* tolerance to high dietary sucrose. We further explored some of the specific metabolic abnormalities that can result when these genes’ activities are reduced in flies, focusing on a gene we call *dHHEX* (*CG7056*), the fly ortholog of human *HHEX*.

## Background

Type 2 diabetes (T2D), a disease state characterized by impaired insulin sensitivity and hyperglycemia, is one of the world’s leading causes of mortality and morbidity [1-3]. In recent years, genome-wide association studies (GWAS) have had success in identifying susceptibility loci for type 2 diabetes and related traits in humans [4,5]. These studies establish associations between markers, such as single nucleotide polymorphisms (SNPs), and disease. However, they typically lack the resolution needed to identify causal variants, because SNPs may exist in linkage disequilibrium with multiple protein-coding loci as well as with non-coding gene-regulatory elements that can act over a long distance [6,7]. Mouse models of diabetes and obesity can serve as convenient platforms to functionally probe a small number of candidate genes [8], but this approach is expensive and slow, limiting the number of genes that can be readily assessed.

The biochemical pathways involved in growth and metabolism are ancient and well conserved across the animal kingdom from *C. elegans* and *Drosophila* to rodents and humans [9]. Analogous to insulin and glucagon in vertebrates, *Drosophila* insulin-like peptides (dILPs) and adipokinetic hormone (Akh) regulate circulating glucose homeostasis. In addition, many tissues known to be important in type 2 diabetes have functional analogs in *Drosophila* including blood, adipose tissue and liver, skeletal muscle, pancreatic beta cells, brain, and kidney [9]. Indeed, *Drosophila melanogaster* raised on diets high in sugar exhibit hallmark features of type 2 diabetes including insulin resistance, fasting hyperglycemia, and increased fat storage [10].

Off-the-shelf genetic tools in *Drosophila*, including mutations and inducible RNA interference (RNAi), allow the functions of specific genes to be rapidly queried; a *Drosophila* genetic approach has recently been used to follow up a small-scale GWAS for Alzheimer pathology [11]. Here we make use of the advantages of *Drosophila* as a model system for exploration of whole-animal metabolism. Starting from a subset of previously published human SNPs and regions associated with disease risk, we utilize *Drosophila* to screen candidate genes in each region in an unbiased manner. We provide functional evidence that disruption of some of these genes can predispose the flies to dietary sucrose-induced lethality. We show in one region that *HHEX* may contribute to type 2 diabetes phenotypes including hyperglycemia and insulin insensitivity; in addition, our data suggests two neighboring genes may also contribute to the risk identified by GWAS. Thus, in addition to implicating specific genes as disease drivers, our work demonstrates the power of *Drosophila* to provide rapid, functional, diet- and environment-sensitive assays for GWAS follow-up studies and to deconvolute regions that contain multiple risk loci.

## Results and discussion

### Fly orthologs of human genes in disease risk-associated regions

We focused on a set of 38 human genomic regions in which SNPs have been associated with type 2 diabetes disease status [12-14] as well as related quantitative traits (QTs), including levels of fasting blood glucose [15,16], triglycerides [17-19], low-density lipoprotein (LDL) [17,18], and high-density lipoprotein (HDL) [17,18]. We included the latter SNPs since there is considerable overlap between mechanisms that regulate lipid and glucose metabolism. Beginning with the 130 human genes located within approximately 100 kb of each SNP, we identified fly orthologs as inferred by Ensembl’s phylogenetic analyses (release 43) [20,21] and found that 71 of the 130 candidate human genes—within 33 of the 38 human genomic regions—have fly orthologs with one-to-one, one-to-many, many-to-one, or many-to-many orthology relationships. In total, we identified 83 fly orthologs corresponding to 71 human genes under consideration. Human GWAS traits, associated index SNPs, genomic regions, candidate genes, and fly orthologs are listed in Table 1 and Additional file 1: Table S1.

**Table 1:** Human genes identified by GWAS

**Region**	**Trait**	**Genes**
1p32.3	LDL	*TMEM61*, *BSND*, *PCSK9*, *USP24*
1p31.3	TG	ANGPTL3*, *DOCK7*, ATG4C**
1p13.3	LDL	*KIAA1324*, *SARS*, CELSR2**, *PSRC1*, *MYBPHL*, *SORT1*
1p12	T2D	*ADAM30*, NOTCH2
1q42.13	TG, HDL	GALNT2
2p24.1	LDL	*APOB*
2p23.3	T2D	NRBP1**, *KRTCAP3*, IFT172, *FNDC4*, *GCKR*, *C2orf16*, *ZNF512*
2p21	T2D	THADA**, PLEKHH2
2q24.3	FG	NOSTRIN*, *SPC25*, G6PC2, ABCB11**
3p25.2	T2D	PPARG**
3p14.1	T2D	ADAMTS9*
3q27.3	T2D	IGF2BP2*, *C3orf65*
5q13.3	LDL	HMGCR, COL4A3BP
6p22.3	T2D	CDKAL1
6p21.32	LDL	*HLA-DPA1*, *COL11A2*, RXRB**, SLC39A7, HSD17B8, *MIR219-1*, RING1**, VPS52, RPS18
7p15.2	T2D	JAZF1
7p13	FG	*AEBP1*, *MIR4649*, *POLD2*, *MYL7*, GCK**, *YKT6*, *CAMK2B*
7q11.23	TG	BAZ1B, BCL7B, *TBL2*, MLXIPL**, *VPS37D*, DNAJC30*, WBSCR22, *STX1A*
8p21.3	TG, HDL	LPL**
8q24.11	T2D	SLC30A8*
8q24.13	TG	TRIB1*
9p21.3	T2D	*MTAP*, *CDKN2A*, *CDKN2B*
9q31.1	HDL	*ABCA1*
10p13	T2D	*NUDT5*, *CDC123*, *CAMK1D*
10q23.33	T2D	IDE*, KIF11*, HHEX**
10q25.2	T2D	TCF7L2**
11p15.1	T2D	NUCB2*, *NCR3LG1*, KCNJ11**, ABCC8*
11p11.2	T2D	EXT2, *ALX4*
11q23.3	TG	*BUD13*, ZNF259, *APOA5*, *APOA4*, *APOC3*, *APOA1*, *SIK3*
12q15	T2D	TSPAN8, *LGR5*
12q23.3	HDL	*MYO1H*, KCTD10*, *UBE3B*, *MMAB*, MVK**
15q21.3	HDL	LIPC**
16q12.1	T2D	*RPGRIP1L*, *FTO*
16q12.2	HDL	*CETP*, *NLRC5*, SLC12A3**, HERPUD1, *MIR138-2*
18q21.1	HDL	LIPG**, ACAA2
19p13.2	LDL	SMARCA4, *LDLR*, *SPC24*, *KANK2*
19p13.11	TG, LDL	GATAD2A, TSSK6, NDUFA13**, *YJEFN3*, *CILP2*, PBX4, *LPAR2*, *GMIP*, ATP13A1**
19q13.33	LDL	*BCL3*, *CBC*, *BCAM*, PVRL2, TOMM40**, *APOE*, *APOC1*, *APOC4*, *APOC2*, CLPTM1, RELB**

List of all human genes located near the SNPs considered in the study. Asterisks indicate genes with *Drosophila* orthologs that function in sucrose tolerance in *Drosophila*; doubled asterisks indicate strong hits. Italicized genes were not evaluated in the *Drosophila* sucrose-intolerance screen, mostly because they lack *Drosophila* orthologs. References and more detailed experimental results are in Additional file 1: Table S1.

### Screen for modifiers of sucrose tolerance

We and others have previously shown that flies fed high-sugar diets, including a 1.0 M sucrose diet, exhibit diabetes-like phenotypes [10,22]. Additionally, flies die as larvae when fed very high levels of dietary sucrose (above 1.25 M), but survival to pupariation is comparable between flies fed 0.15 M and 1.0 M sucrose diets (Additional file 1: Figure S1). We hypothesized that knocking down a gene that mediates sucrose tolerance would affect larval viability differently on high- vs. low-sucrose diets. Such a gene would be required for survival to pupariation on a 1.0 M sucrose diet, but may prove dispensable on a control 0.15 M sucrose diet.

**Figure 1:**
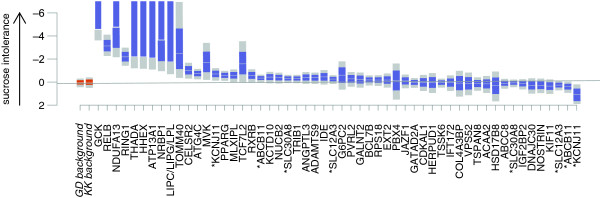
**Effect of sucrose on survival of RNAi candidates.** Ranked estimates and confidence intervals (orange/purple: 95%, gray: Bonferroni-adjusted, N = 113) for the ln(OR) of knockdown vs. non-knockdown sibling pupariation on 1.0 M sucrose vs. 0.15 M sucrose. For each human gene, the cross with the most dramatic effect is shown; crosses that were lethal independent of dietary sucrose are omitted. Bars are labeled with the names of the human orthologs corresponding to each RNAi line; the names of the RNAi lines themselves are in Additional file 1: Table S1. We considered crosses whose 95% confidence intervals exclude zero as hits, and we considered crosses whose Bonferroni-adjusted confidence intervals exclude zero as strong hits. In a few cases, a gene was a hit in both directions; in such cases, both crosses are shown and the gene name is marked with an asterisk. More complete details are in Additional file 1: Tables S1 and S2.

**Figure 2:**
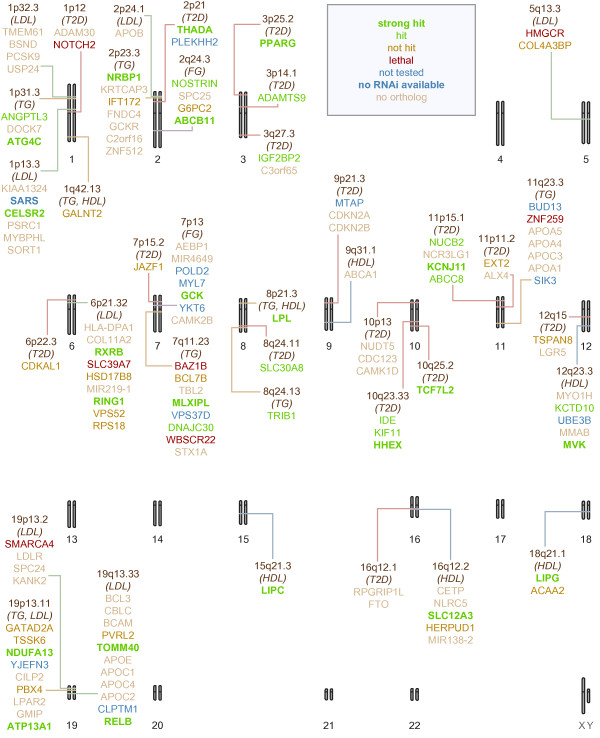
**Graphical summary of the *Drosophila *sucrose-intolerance screen.** The 38 regions of interest, and the human genes located in them, are marked on a schematic karyogram of the human genome. The regions are labeled with the metabolic traits with which they are associated. The gene names are color-coded to indicate their outcome in our screen. More complete details are in Additional file 1: Table S1.

To test this hypothesis and to identify modifiers of the sucrose intolerance phenotype, we screened knockdowns of the selected genes (Table 1) using the now-classic GAL4/UAS system [23]. Fly lines containing inducible RNA-interference (*UAS-RNAi*) elements were acquired for most of these genes; multiple fly lines were available for many loci, and a total of 137 RNAi fly lines were tested. We used *tub*_*P*_*-GAL4* to direct broad expression of GAL4, which in turn induces broad expression of the RNAi-encoding transgene. Sucrose tolerance was then assessed by (i) scoring pupariation rates relative to non-RNAi controls and (ii) comparing high-sucrose vs. low-sucrose feeding. An important strength of this approach is that we can distinguish between sucrose-dependent and sucrose-independent toxicity. The results of the screen are detailed in Additional file 1: Table S1 and summarized in Figures 1 and 2 and Table 1.

We defined hits as crosses in which knockdown resulted in statistically significant sucrose-sensitive toxicity at a 0.05 threshold. We defined strong hits as results that had *p*-values less than 4.42 × 10^–4^, corresponding to a Bonferroni adjustment for the total of 113 lines for which sucrose tolerance could be assessed (we excluded crosses where sucrose tolerance could not be assessed due to sucrose-independent lethality). 47 of the screened lines were hits, corresponding to 34 human genes; 29 of these were strong hits, corresponding to 22 human genes. As a control, we verified that the relative survival to pupariation was similar for flies fed 1.0 M sucrose vs. 0.15 M sucrose food for the GD and KK genetic backgrounds (without RNAi transgenes) used in the study (Additional file 1: Table S2, Figure 1).

### Regions containing a single candidate gene

The approximately 100 kb radius window we applied included only a single human gene at 12 regions. At each of these regions, it seems plausible that variants in this single gene are causative for the associated traits. 10 of these regions were tested with fly orthologs (two regions did not contain fly orthologs). Seven fly orthologs were identified as hits in at least one cross. Strong hits corresponded to *PPARG* and *TCF7L2* (T2D candidates), *LIPC* (HDL candidate), and *LPL* (TG and HDL candidate); orthologs of *ADAMTS9* and *SLC30A8* (T2D candidates) and *TRIB1* (TG candidate) were also hits. The 7/10 hit rate at single-gene regions indicates that our model likely reflects at least some important aspects of human T2D and related QTs, and that our assay can provide useful modifier data.

Not all single-gene regions tested positive in our *Drosophila* assay: orthologs of *GALNT2*, *CDKAL1*, and *JAZF1* failed to display modifier activity (Figures 1 and 2, Table 1, Additional file 1: Table S1). This may be due to several factors. First, the RNAi constructs and insertions used work with differing efficacy, at least in part likely due to their random insertion sites within the genome [24]. Consistent with this phenomenon, some RNAi lines targeting the same *Drosophila* gene gave discordant results in our assay; most commonly, one line showed modifier activity and another did not. Second, phylogenetic inference of orthology may not be correct. Indeed, Ensembl’s inferences were refined while our study was underway. Third, the GWAS result may be a false positive, or the true causative variant may lie outside of the window we initially selected or may be undetected within the region. However, *GALNT2* acts in metabolic pathways [25] and a coding mutation in *CDKAL1* has been closely correlated with T2D risk in humans [26]. Fourth, our screening approach may not identify loci that are risk factors due to upregulation. Lastly, failure of *Drosophila* to confirm modifier status for several of these regions may reflect limitations of using flies to explore GWAS.

### Regions containing multiple candidate genes

At the remaining regions, our approximately 100 kb radius window defined more than one candidate human gene, many with fly orthologs. The region near *rs4607517* contains the gene *GCK*, encoding glucokinase, which is required for glucose-stimulated insulin secretion and proper glucose metabolism. *GCK* mutations are causative alleles in a monogenic form of diabetes [27], making it a strong candidate to further validate our approach. Indeed, sucrose-specific toxicity was strongly enhanced by knockdown of all but one of the four putative fly orthologs of *GCK* (Figure 1, Additional file 1: Table S1).

For other regions we tested orthologs of multiple human genes, and at six of the remaining regions our sucrose toxicity screen implicated a single human gene ortholog. These genes were *THADA* and *IGF2BP2* (T2D candidates), *CELSR2* (LDL candidate), *NRBP1* (TG candidate), *SLC12A3* and *LIPG* (HDL candidates). At six regions our screen did not identify any hits. While this may be explained in part by potential lack of sensitivity in our system, in all cases these regions included other genes we were not able to test.

At nine regions, our screen implicated more than one human gene. This may reflect a lack of specificity of this assay, perhaps due to off-target effects of the RNAi constructs or differences in insect and mammalian physiology. On the other hand, these results highlight the fact that model organism screening has the ability to identify multiple modifier loci within a single region and at the level of individual genes. Of particular utility, genes that are tightly linked in humans can be subjected to individual functional testing using specific RNAi knockdown. A striking example of this is a region at 10q23.33 that contains *IDE*, *KIF11*, and *HHEX*, three genes with clear one-to-one fly orthologs. Genome-wide scans [12,13,28] have consistently reported SNP signals associated with T2D near this region. Attention has focused on *HHEX*, which has been most closely linked with these signals [29], and which encodes a metabolism-related HOX-class transcription factor. Indeed, our screening identified *CG7056*—which we refer to here as *dHHEX*—as the most robust modifier of sucrose-mediated lethality in our study (Figure 1, Additional file 1: Table S1).

We also identified *Drosophila* orthologs of the neighboring genes *IDE* and *KIF11*—*Drosophila* genes *ide* and *klp61F*—as modifiers (Figures 1 and 2, Additional file 1: Table S1). Intriguingly, *IDE* knockout mice exhibit hyperglycemia and insulin insensitivity in an age-dependent manner [30,31]. *KIF11* has also been knocked out in mice but is embryonic lethal; metabolic effects of partial loss of *KIF11* have not been characterized [32]. Our data suggest that *IDE* and *KIF11* may contribute to patients’ metabolic risk. Their role may be obscured by the high risk conferred by neighboring *HHEX* locus; the three loci may act independently, or perhaps variations in copy number affect the three loci to increase patient risk.

### Further characterization of *dHHEX*: diet

**Figure 3:**
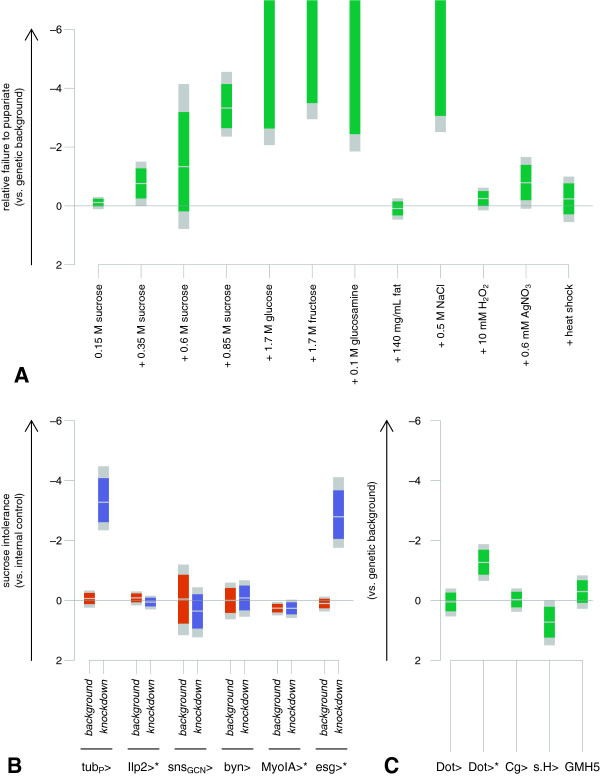
**Diet-specific and tissue-specific lethality of dHHEX knockdown. A**. *tub*_*P*_*>RNAi*^*dHHEX-V15721 *^flies tolerate many non-ideal diets, but are sensitive to high sucrose, as well as to glucosamine and to high salt. Estimates and confidence intervals (green: 95%, gray: Bonferroni-adjusted, N = 12) of ln(OR) are shown comparing odds of knockdown vs. non-knockdown animals pupariating compared to the genetic background control on that diet. More complete details are in Additional file 1: Table S3. **B**–**C**. *tub*_*P*_*-GAL4*, *Dot-GAL4*, and *esg-GAL4* driving dHHEX knockdown using *UAS-RNAi*^*dHHEX-V15721*^ confer lethality on high sucrose, but a number of other drivers do not. Estimates and confidence intervals (colored: 95%, gray: Bonferroni-adjusted, N = 11) of ln(OR) are shown for survival of knockdown vs. non-knockdown animals, compared to the genetic background control. Pupariation (unmarked) or eclosion (asterisks) or both were scored. More complete details are in Additional file 1: Tables S4 and S5.

We next used *Drosophila* to further explore aspects of *dHHEX*’s role in the response to high dietary sucrose. Raised on a variety of feeding conditions (Additional file 1: Table S3, Figure 3A) *tub*_*P*_*>RNAi*^*dHHEX*^ flies remained comparable to wild type on a number of stressful diets including diets containing hydrogen peroxide and silver nitrate and, notably, a high-fat diet. We observed elevated lethality when *tub*_*P*_*>RNAi*^*dHHEX*^ flies were raised on high-salt diets (slightly hyperosmolar relative to 1.0 M sucrose food). This result suggests an impaired ability to respond to hyperosmolar conditions that may contribute to sucrose-dependent lethality. To better understand the role of *dHHEX* specifically in glucose metabolism, we used dietary glucosamine to explore the hexosamine biosynthetic pathway (HBP), a primary pathway of glucose metabolism. The HBP has been implicated in mechanisms of glucose toxicity [33] and dietary glucosamine increases HBP flux in flies [34]. *tub*_*P*_*>RNAi*^*dHHEX*^ flies proved glucosamine-intolerant: the addition of 0.1 M glucosamine to a control diet resulted in failure to pupariate. The ability of low levels of glucosamine to cause lethality suggests that glucose metabolism is indeed a primary effector of dietary sucrose toxicity in *tub*_*P*_*>RNAi*^*dHHEX*^ flies.

### Further characterization of *dHHEX*: tissues

We next used targeted knockdown to determine which tissues require normal *dHHEX* activity in the face of high dietary sucrose (Additional file 1: Table S4, Figure 3B–C). Mammalian studies have implicated HHEX function in diverse tissues and organs including liver, heart, pancreas, thyroid, and hematopoietic cells [35-39]. We used a panel of tissue-selective GAL4 lines to direct expression of the *UAS-RNAi*^*dHHEX*^ transgene in specific tissues: *dilp2-GAL4* targets insulin producing cells (beta cell analogs), *Cg-GAL4* targets several tissues including the fat body (functional analog of mammalian liver and adipose tissue), *Dot-GAL4* targets several tissues including nephrocytes (functional analogs of glomerular podocytes) and gut, *GMH5* targets heart, *srp.Hemo-GAL4* targets hemocytes (phagocytic blood cell analogs) as well as nephrocytes, *sns*_*GCN*_*-GAL4* targets nephrocytes, *esg-GAL4* targets several cell types including midgut stem cells, *MyoIA-GAL4* targets differentiated midgut, and *byn-GAL4* targets hindgut. Flies carrying a GAL4 driver plus *UAS-RNAi*^*dHHEX*^ were raised on high-sucrose diets along with control animals, and the number of knockdown to non-knockdown animals surviving to pupariation or eclosion on high or low sucrose was scored. Of the three distinct *UAS-RNAi*^*dHHEX*^ lines we used in the initial screen, we focused on the V15721 line, which exhibited the most survival of knockdown flies on 0.15 M sucrose when driven by *tub*_*P*_*-GAL4*.

Knockdown of *dHHEX* in the heart, hemocytes, nephrocytes, hindgut, and differentiated midgut did not affect viability on high-sucrose feeding compared to low-sucrose feeding, and perhaps surprisingly, neither did knockdown in the insulin producing cells or the fat body (Figure 3B–C). However, knockdown driven by either *Dot-GAL4* or *esg-GAL4* did affect survival to eclosion in a sugar-sensitive manner (although, interestingly, knockdown driven by *Dot-GAL4* did not affect survival to pupariation). Both of these drivers express in a range of tissues but one point of overlap in their domains is in the midgut stem cells.

### Further characterization of *dHHEX*: metabolics

Given the fat body’s important role in metabolism, we profiled hemolymph (blood) glucose, body size, and whole-animal triglyceride levels in *Cg>RNAi*^*dHHEX-V15721*^ wandering third-instar and adult flies reared on 1.0 M sucrose. Dicer-2 (Dcr-2) overexpression was included to enhance RNAi efficacy [24]. Female flies were studied because this experimental genotype is male-lethal.

**Figure 4:**
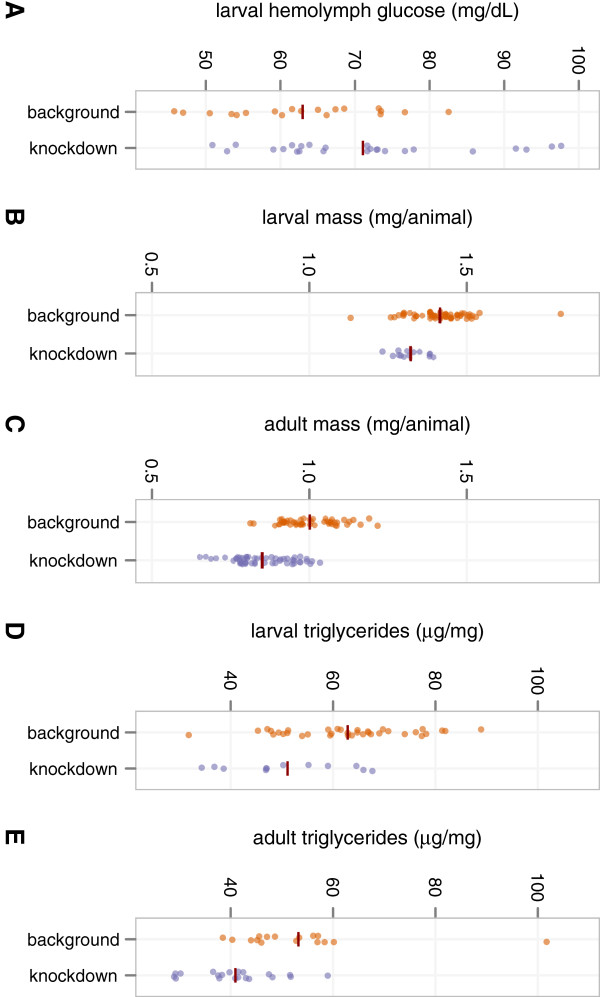
**Metabolic profiling of Cg>RNAi**^**dHHEX-V15721**^**, Dcr-2. A**. Wandering third-instar females of *Cg>RNAi*^*dHHEX-V15721*^*, Dcr-2* are hyperglycemic. Each measurement is made from a pooled sample of hemolymph from 5–8 animals; dark red line segments show mean values. *p* < 0.029. **B**–**C**. Wandering third-instar and newly eclosed adult females of *Cg>RNAi*^*dHHEX-V15721*^*, Dcr-2* have reduced body size. Each measurement is the mean per-animal mass of a group of 6–10 animals; dark red line segments show mean values. *p* < 0.001 for both larvae and adults. **D**–**E**. Wandering third-instar and newly eclosed adult females of *Cg>RNAi*^*dHHEX-V15721*^*, Dcr-2* have reduced triglyceride levels. Each measurement is made from a pooled sample of whole-animal homogenate from 6–10 animals; dark red line segments show mean values. *p* < 0.007 for both larvae and adults (*p* < 0.002 for adults when one extreme outlier is excluded). All *p*-values for metabolic data were calculated using a bidirectional *t*-test without assuming equal variances. More complete details are in Additional file 1: Tables S6–S8.

Both larval and adult *Cg>RNAi*^*dHHEX-V15721*^*, Dcr-2* flies were significantly smaller than controls when reared on a high-sucrose diet throughout development (*p* < 0.001, Additional file 1: Table S6, Figure 4B–C). Since flies have a single receptor that is orthologous to both human insulin and human IGF1 receptors, and since this receptor controls both glucose homeostasis and growth [40-42], the smaller body size of knockdown animals suggests that *Cg>RNAi*^*dHHEX-V15721*^*, Dcr-2* flies have decreased insulin signaling activity and hence increased insulin resistance when confronted with high dietary sucrose. Consistent with this view, wandering (non-feeding) *Cg>RNAi*^*dHHEX-V15721*^*, Dcr-2* larvae were hyperglycemic after high-dietary-sucrose rearing (*p* < 0.029, Additional file 1: Table S6, Figure 4A). Intriguingly, in both *Cg>RNAi*^*dHHEX-V15721*^*, Dcr-2* larvae and adults, hyperglycemia and reduced body size were accompanied by significantly lower triglyceride levels when compared to controls (*p* < 0.007, Additional file 1: Table S6, Figure 4D–E).

## Conclusions

To date, genome-wide scans for genes and alleles that contribute to metabolic disease risk have identified numerous candidates, but functional follow-up studies have been more difficult to perform in mammalian systems. We hypothesized that human genes involved in susceptibility to developing T2D and related traits can be prioritized by knocking down their fly orthologs and assaying sucrose-sensitive lethality. Evidence in support of our hypothesis includes the fact that this assay identifies *GCK*, a gene known to cause a monogenic form of diabetes, and the fact that sucrose-sensitive lethality correlates with metabolic abnormalities in the flies. To our knowledge, this is the first large-scale functional study of metabolic-trait candidate genes identified by GWAS analysis, and the first to specifically address an interaction between genes and environment.

Through more detailed analysis of the function of the *Drosophila HHEX* ortholog, we have shown that this gene plays an important role in whole-animal metabolism in this system through its effects in the fat body—a functional analog of mammalian liver and adipose tissue. Loss of *dHHEX* results in insulin resistance and hyperglycemia and, interestingly, a reduction of whole-animal triglyceride levels in this system. It has been proposed that the conversion of fatty acids into triglycerides may protect against tissue lipotoxicity [43]; the hyperglycemia observed in *Cg>RNAi*^*dHHEX-V15721*^ flies suggests *dHHEX* may play a role in determining the capacity of the fly to store energy as triglycerides. We additionally showed that there are multiple other candidate genes for T2D and related QTs (fasting glucose, triglycerides, LDL, and HDL) that have diet-dependent roles in overall organismal viability. Further systematic study of these genes, including T2D candidate genes such as *PPARG*, *IDE*, and *KIF11*, may help elucidate their molecular functions in their respective pathways. Since many fundamental aspects of metabolism have been conserved during evolution, it is reasonable to hypothesize that these functions may be similar in humans as in flies; whether this is true will, of course, have to be determined case by case.

In general, *Drosophila* offers a rich resource for providing rapid, inexpensive, whole-animal tests of gene function. In addition to screening candidate genes identified by GWAS approaches, this same approach could prove useful, as whole genome sequencing becomes more common, for identifying specific mutations that are causative rather than simply correlated. Perhaps its most important advantage is the ability to assess all candidates in an unbiased manner, identifying surprising hits and untangling complex regions.

## Methods

### Fly stocks

RNAi stocks (listed in Additional file 1: Table S1) were acquired from the Vienna Drosophila Resource Center, as well as genetic background controls *w*^*1118*^ (for GD lines, VDRC #60000) and *y*^*–*^ *w*^*1118*^*; P{attP, y*^*+*^*, w*^*3’*^*}VIE-260B* (for KK lines, VDRC #60100) [24]. *tub*_*P*_*-GAL4* (BDSC #5138) [44], *Cg-GAL4* (BDSC #7011) [45], *dilp2-GAL4* (BDSC #37516) [46], *esg-GAL4* (BDSC #26816), *Dot-GAL4* (BDSC #6903) [47,48], *UAS-Dcr-2* (BDSC #24648) [24], and *TM6B, Tb*^*1*^ (BDSC #120) are available from the Bloomington Drosophila Stock Center. *MyoIA-GAL4* (DGRC-K #112001) is available from the Drosophila Genetic Resource Center, Kyoto. Additional fly stocks were generously provided by the *Drosophila* community: *GMH5* by Rolf Bodmer [49], *sns*_*GCN*_*-GAL4* by Susan Abmayr [50], *srp.Hemo-GAL4* by Katja Brückner [51,52], *byn-GAL4* by Volker Hartenstein [53,54], and a *T(2;3)* balancer by Larry Zipursky.

### Fly media

We modified a commonly used *Drosophila* semi-defined medium [55] as previously described [10]. Briefly, we replaced all added sugars in the recipe (glucose and sucrose) with 51.3 g/L sucrose (to yield 0.15 M sucrose) plus any other desired components. The primary screen was carried out on 0.15 M sucrose (low sucrose) and 1.0 M sucrose (high sucrose) foods.

### Scoring and statistics

In *tub*_*P*_*-GAL4*, *byn-GAL4*, and *sns*_*GCN*_*-GAL4* studies, flies carrying a GAL4-encoding transgene and a balancer as the homologous chromosome were crossed to flies carrying a RNAi-encoding transgene, and survival to pupariation was scored by counting non-tubby (driver*>RNAi*) and tubby (*UAS-RNAi; TM6B* or a *T(2;3)* balancer) pupae, except for a small number of exceptions, where survival to adulthood of non-curly (*tub*_*P*_*>RNAi*) compared to curly (*tub*_*P*_*-GAL4; CyO*) or non-stubble (*tub*_*P*_*>RNAi*) compared to stubble (*tub*_*P*_*-GAL4; TM3, Sb*^*–*^) animals was scored instead. In studies of other drivers, either a similar cross was performed and survival to adulthood was scored by counting non-curly (driver*>RNAi*) and curly (driver; *CyO*) animals, or else rates of eclosion were compared for a fixed number of knockdown embryos compared to a fixed number of control embryos possessing the *GAL4* insertion and the proper genetic background, but lacking the RNAi-encoding insertion. Counts that were extremely different from experimental replicates were excluded from analysis (3 out of over 800 replicates were excluded in this way). If fewer than 5 knockdown animals survived in all experimental replicates for a given comparison, then we considered the knockdown to be generally toxic and did not assess sucrose intolerance.

For each comparison, we used Fisher’s exact test to assess whether our data were consistent with the null hypothesis that relative survival of knockdown flies was the same on high- and low-sucrose feeding, as well as to compute point estimates and confidence intervals for the natural logarithm of the odds ratio (ln(OR)) for survival of the knockdown and control genotypes on high and low sucrose. We considered crosses to be hits when statistically significant with a 0.05 threshold, and we considered crosses to be strong hits when statistically significant with a Bonferroni-adjusted threshold. For the RNAi screen for sucrose sensitivity, this threshold was 4.42 × 10^–4^, corresponding to 113 crosses tested (excluding crosses that were lethal on both diets, since no determination about sucrose sensitivity can be made for these crosses). For the diet survey, this threshold was 4.17 × 10^–3^, corresponding to 12 diets tested. For the driver survey, this threshold was 4.55 × 10^–3^, corresponding to 11 driver-phenotype pairs tested. We used confidence intervals for hypothesis testing; they could in principle also be used for effect-size comparison and equivalence testing.

Metabolic parameters were comparing using two-sided unpaired *t*-tests without assuming equal variances.

Computations were performed in R, a language and environment for statistical computing, version 2.15.1. Plots were generated in R, some using the ggplot2 package. Some tables were constructed using XeLaTeX and the longtable and booktabs packages.

### Metabolic studies

Hemolymph glucose and whole-animal triglycerides were measured as previously described [10]. Briefly, to collect hemolymph, wandering third-instar larvae were lanced and hemolymph from 5–8 larvae was pooled to collect 1 μL. Glucose levels were measured using the Infinity Glucose Hexokinase Reagent kit (Thermo Fisher #TR15421). Triglycerides were measured using the Infinity Triglycerides Reagent kit (Thermo Fisher #TR22321) on whole-animal homogenates of groups of 6–10 animals. Per-animal mass was measured by weighing groups of 6–10 animals.

## Abbreviations

GWAS: Genome-wide association study; HBP: Hexosamine biosynthesis pathway; HDL: High-density lipoprotein; LDL: Low-density lipoprotein; QT: Quantitative trait; SNP: Single nucleotide polymorphism; T2D: Type 2 diabetes.

## Competing interests

RLC and TJB are co-founders of Medros, Inc., which uses models of human disease for drug development.

## Authors’ contributions

RLC, TJB, and FSC conceived of the project, and NN and FSC identified human and fly genes of interest. All authors contributed to the design of the experiments. PVR conducted hemolymph glucose measurements, JLF conducted mass and triglyceride measurements, and JP and JN conducted all other experiments. JP designed and conducted the statistical analysis. JP, PVR, NN, and TJB wrote this manuscript, all authors contributed to completing and revising it, and all authors read and approved the final manuscript.

## Supplementary Material

Additional file 1 Includes Figure S1 and Tables S1–S8. Click here for file
