# Supplementary material for: A Drosophila functional evaluation of candidates from human genome-wide association studies of type 2 diabetes and related metabolic traits identifies tissue-specific roles for dHHEX
Source: BMC Genomics. 2013 Feb 27;14:136. doi: 10.1186/1471-2164-14-136 (PMC3608171; doi:10.1186/1471-2164-14-136)
Supplement: Additional file 1 — Includes Figure S1 and Tables S1–S8. [file 1471-2164-14-136-S1.pdf]

Supplementary material for

Pendse J, Ramachandran PV, Na J, Narisu N, Fink JL, Cagan RL, Collins FS, and Baranski TJ:
A Drosophila functional evaluation of candidates from human genome-wide association studies of type 2 diabetes and related metabolic traits identifies tissue-specific roles for dHHEX.

Figure S1. Wild type flies tolerate up to 1.25 M dietary sucrose.

Table S1. RNAi screen for sucrose sensitivity: knockdowns.

Table S2. RNAi screen for sucrose sensitivity: controls.

Table S3. Diet survey. Ubiquitous dHHEX knockdown results in intolerance to sugar and salt but not other stressors.

Table S4. Driver survey, part 1. Knockdown of dHHEX in the tubP or esg domains results in sucrose intolerance.

Table S5. Driver survey, part 2. Knockdown of dHHEX in the Dot domain results in sucrose intolerance.

Table S6. Cg>RNAi$^{dHHEX\text{-}V15721}$, Dcr-2 larvae are hyperglycemic.

Table S7. Cg>RNAi$^{dHHEX\text{-}V15721}$, Dcr-2 flies have reduced body mass.

Table S8. Cg>RNAi$^{dHHEX\text{-}V15721}$, Dcr-2 flies have reduced triglyceride levels.

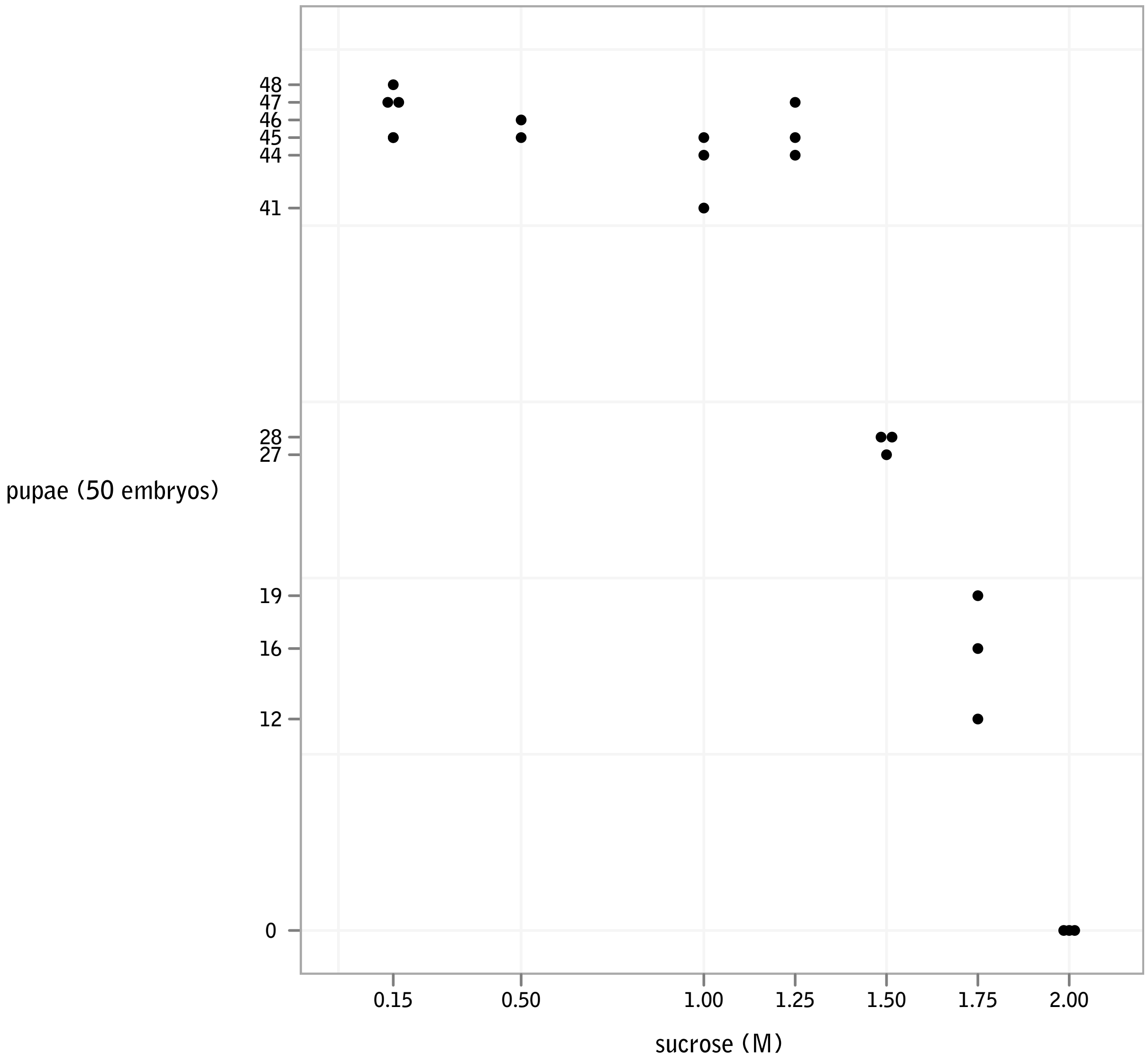

Figure S1. Wild type flies tolerate up to 1.25 M dietary sucrose. Survival to pupariation of w^{1118} flies reared with different levels of dietary sucrose, starting from 50 embryos per trial. Survival is comparable on 0.15 M, 1.0 M, and 1.25 M sucrose, but it decreases in a dose-dependent manner on 1.5 M and 1.75 M sucrose, and no pupae are seen on 2.0 M sucrose.

Table S1 – RNAi screen
(data associated with Figures 1–2)

				scoring: tub$_{P}$ > RNAi					confidence interval bounds				
				1.0 M sucrose		0.15 M sucrose			95%		Bonferroni		
**Locus**	**Human Gene**	**Fly Gene**	**Insertion**	RNAi	control	RNAi	control	**ln(OR)**	lower	upper	lower	upper	**p-value**
**1p32.3**	**TMEM61**												
low-density lipoprotein	(no ortholog)												
Willer: rs11206510													
Kathiresan: rs11591147	**BSND**												
	(no ortholog)												
	**PCSK9**												
	(no ortholog)												
	**USP24**												
	(no ortholog)												
**1p31.3**	**ANGPTL3**	**CG9593**	**V24164**	**108**	**110**	**112**	**104**	**–0.0922**	–0.4866	0.3016	–0.7862	0.5999	**6.3276E–01**
triglyceride	(many-to-one ortholog)			28	21	25	23						
Willer: rs1748195				72	80	50	41						
Kathiresan: rs12130333				8	9	37	40						
			**V24165**	**154**	**228**	**310**	**319**	**–0.3634**	–0.6298	–0.0984	–0.8360	0.1040	**6.2409E–03**
				29	48	48	47						
				82	119	103	103						
				43	61	159	169						
			**V105528**	**400**	**490**	**301**	**289**	**–0.2434**	–0.4577	–0.0295	–0.6235	0.1355	**2.2373E–02**
				144	187	66	65						
				78	125	103	96						
				178	178	132	128						
	**DOCK7**												
	(no ortholog)												
	**ATG4C**	**CG6194**	**V22293**	**109**	**130**	**175**	**284**	**0.3075**	–0.0218	0.6367	–0.2737	0.8880	**6.1945E–02**
	(many-to-one ortholog)			26	21	41	57						
				51	59	68	114						
				32	50	66	113						
			**V22294**	**167**	**315**	**557**	**496**	**–0.7501**	–0.9808	–0.5216	–1.1610	–0.3473	**3.2261E–11**
				74	141	79	95						
				48	71	161	125						
				45	103	317	276						
**1p13.3**	**KIAA1324**												
low-density lipoprotein	(no ortholog)												
Willer: rs599839													
Kathiresan: rs646776	**SARS**	**CG17259**	no RNAi										
	(one-to-one ortholog)												

continued on next page

				scoring: $tub_P >$ RNAi					confidence interval bounds				
				1.0 M sucrose		0.15 M sucrose			95%		Bonferroni		
**Locus**	**Human Gene**	**Fly Gene**	**Insertion**	RNAi	control	RNAi	control	**ln(OR)**	lower	upper	lower	upper	**p-value**
	**CELSR2**	**CG11895**	**V1665**	**83**	**66**	**145**	**163**	**0.3454**	–0.0657	0.7593	–0.3751	1.0757	**9.0359E–02**
	(many-to-one ortholog)	starry night		32	20	59	66						
				32	28	53	58						
				19	18	33	39						
			**V51379**	**153**	**221**	**118**	**95**	**–0.5835**	–0.9390	–0.2303	–1.2119	0.0367	**7.8596E–04**
				34	55	19	18						
				37	70	53	20						
				82	96	46	57						
			**V51382**	**234**	**308**	**146**	**181**	**–0.0598**	–0.3457	0.2265	–0.5645	0.4466	**6.7268E–01**
				50	62	37	45						
				58	79	35	42						
				126	167	74	94						
			**V107993**	**62**	**358**	**116**	**236**	**–1.0418**	–1.4102	–0.6803	–1.6981	–0.4109	**2.4612E–09**
				21	66	22	52						
				21	126	42	102						
				20	166	52	82						
	**PSRC1**												
	(no ortholog)												
	**MYBPHL**												
	(no ortholog)												
	**SORT1**												
	(no ortholog)												
**1p12**	**ADAM30**												
type 2 diabetes	(no ortholog)												
Zeggini: rs10923931													
	**NOTCH2**	**CG3936**	**V100002**	**0**	**291**	**1**	**211**	**toxic knockdown**					
	(many-to-one ortholog)	Notch		0	139	0	62						
				0	68	1	90						
				0	84	0	59						
**1q42.13**	**GALNT2**	**CG3254**	**V26162**	**105**	**106**	**162**	**165**	**0.0089**	–0.3523	0.3700	–0.6273	0.6450	**1.0000E+00**
triglyceride,	(one-to-one ortholog)	polypeptide GalNAc		30	46	76	82						
high-density lipoprotein		transferase 2		75	60	86	83						
Willer: rs2144300													
Kathiresan: rs4846914			**V26163**	**115**	**104**	**218**	**168**	**–0.1597**	–0.5065	0.1872	–0.7709	0.4518	**3.5103E–01**
				63	42	106	86						
				52	62	112	82						

continued on next page

Locus	Human Gene	Fly Gene	Insertion	scoring: tub_P > RNAi 1.0 M sucrose RNAi	1.0 M sucrose control	0.15 M sucrose RNAi	0.15 M sucrose control	ln(OR)	confidence interval bounds 95% lower	95% upper	Bonferroni lower	Bonferroni upper	p-value
**2p24.1** low-density lipoprotein Willer, Kathiresan: rs693	**APOB** (no ortholog)												
**2p23.3** triglyceride Kathiresan: rs780094	**NRBP1** (many-to-one ortholog)	**CG1098** Madm	**V27346**	**135** 66 14 55	**285** 128 28 129	**316** 55 76 185	**344** 59 98 187	**−0.6617**	−0.9267	−0.3993	−1.1331	−0.2000	**2.8896E−07**
			**V27347**	**361** 130 168 63	**378** 145 160 73	**587** 115 105 367	**520** 109 134 277	**−0.1671**	−0.3580	0.0236	−0.5061	0.1712	**7.8999E−02**
			**V101758**	**1** 0 0 1	**260** 80 77 103	**30** 7 23 0	**200** 54 111 35	**−3.6591**	−7.3682	−1.8468	−∞	−1.0679	**8.0921E−10**
	**KRTCAP3** (no ortholog)												
	**IFT172** (one-to-one ortholog)	**CG13809** osm-1	**V24793**	**73** 50 23	**80** 64 16	**175** 13 82 80	**167** 17 69 81	**−0.1381**	−0.5385	0.2613	−0.8436	0.5636	**4.9701E−01**
			**V24795**	**110** 74 36	**88** 63 25	**210** 12 80 118	**194** 18 80 96	**0.1437**	−0.2118	0.5007	−0.4813	0.7744	**4.3453E−01**
	**FNDC4** (no ortholog)												
	**GCKR** (no ortholog)												
	**C2orf16** (no ortholog)												
	**ZNF512** (no ortholog)												
**2p21** type 2 diabetes Scott: rs7578597	**THADA** (one-to-one ortholog)	**CG15618**	**V40006**	**0** 0 0	**134** 65 69	**57** 23 34	**212** 83 129	**−∞**	−∞	−2.2206	−∞	−1.3286	**1.2392E−11**
	**PLEKHH2** (many-to-one ortholog)	**CG43867**	not tested										

continued on next page

				scoring: tub_P > RNAi					confidence interval bounds				
				1.0 M sucrose		0.15 M sucrose			95%		Bonferroni		
Locus	Human Gene	Fly Gene	Insertion	RNAi	control	RNAi	control	ln(OR)	lower	upper	lower	upper	p-value
**2q24.3**	**NOSTRIN**	**CG42388**	**V26372***	**206**	**172**	**112**	**142**	**0.4170**	0.0851	0.7506	–0.1667	1.0069	**1.1872E–02**
fasting glucose	(one-to-one ortholog)			129	104	44	55						
Chen: rs563694				46	47	68	87						
				31	21								
			**V40328**	**9**	**117**	**23**	**163**	**–0.6049**	–1.5406	0.2467	–2.3273	0.8290	**1.8269E–01**
				5	59	7	50						
				4	58	16	113						
			**V48694**	**0**	**125**	**0**	**312**	**toxic knockdown**					
				0	63	0	136						
				0	62	0	176						
			**V48695**	**0**	**138**	**0**	**280**	**toxic knockdown**					
				0	79	0	115						
				0	59	0	165						
	**SPC25**												
	(no ortholog)												
	**G6PC2**	**CG15400**	**V7261**	**51**	**72**	**32**	**26**	**–0.5494**	–1.2318	0.1247	–1.7471	0.6194	**1.0972E–01**
	(many-to-one ortholog)			23	34	26	14						
				28	38	6	12						
	**ABCB11**	**CG3879**	**V42513**	**494**	**418**	**377**	**409**	**0.2484**	0.0527	0.4443	–0.0985	0.5963	**1.1368E–02**
	(many-to-many ortholog)	Multi drug resistance 49		246	173	86	94						
				105	111	145	168						
				143	134	146	147						
			**V42514**	**502**	**462**	**375**	**373**	**0.0776**	–0.1180	0.2734	–0.2694	0.4250	**4.3571E–01**
				197	164	99	85						
				178	165	138	138						
				127	133	138	150						
		**CG8523**	**V51165**	**0**	**416**	**0**	**420**	**toxic knockdown**					
		Multi drug resistance 50		0	101	0	68						
				0	227	0	175						
				0	88	0	177						
			**V51166**	**256**	**119**	**729**	**587**	**0.5491**	0.3003	0.8017	0.1121	1.0003	**8.0946E–06**
				37	26	167	176						
				112	55	182	193						
				107	38	380	218						
		**CG10181**	**V9019**	**177**	**234**	**233**	**240**	**–0.2493**	–0.5243	0.0250	–0.7361	0.2348	**6.8223E–02**
		Multiple drug resistance 65		20	30	37	35						
				45	50	84	105						
				112	154	112	100						
		**CG10226**	**V108196**	**502**	**716**	**531**	**542**	**–0.3344**	–0.5034	–0.1657	–0.6349	–0.0350	**7.6364E–05**
				92	152	105	99						
				201	252	230	220						
				209	312	196	223						

continued on next page

				scoring: tub_P > RNAi					confidence interval bounds				
				1.0 M sucrose		0.15 M sucrose			95%		Bonferroni		
**Locus**	**Human Gene**	**Fly Gene**	**Insertion**	RNAi	control	RNAi	control	**ln(OR)**	lower	upper	lower	upper	**p-value**
**3p25.2**	**PPARG**	**CG18023**	**V10396**	**197**	**351**	**487**	**482**	**–0.5875**	–0.8094	–0.3672	–0.9823	–0.1986	**7.4639E–08**
type 2 diabetes	(many-to-one ortholog)	Ecdysone-induced protein 78C		85	157	105	114						
Scott: rs1801282				60	112	156	136						
				52	82	226	232						
**3p14.1**	**ADAMTS9**	**CG14869**	**V33346**	**183**	**350**	**231**	**315**	**–0.3380**	–0.5927	–0.0841	–0.7896	0.1103	**7.1741E–03**
type 2 diabetes	(many-to-one ortholog)			57	102	38	64						
Zeggini: rs4607103				66	164	106	153						
				60	84	87	98						
			**V33347**	**70**	**314**	**43**	**240**	**0.2182**	–0.2150	0.6599	–0.5367	1.0040	**3.4741E–01**
				22	105	15	58						
				19	133	17	129						
				29	76	11	53						
**3q27.3**	**IGF2BP2**	**CG1691**	**V20321**	**340**	**256**	**338**	**337**	**0.2806**	0.0531	0.5086	–0.1218	0.6850	**1.3260E–02**
type 2 diabetes	(many-to-one ortholog)	IGF-II mRNA-		107	80	124	112						
Scott: rs4402960		binding protein		99	75	106	109						
				134	101	108	116						
			**V20322**	**329**	**310**	**512**	**457**	**–0.0541**	–0.2591	0.1509	–0.4177	0.3095	**6.1013E–01**
				125	120	216	191						
				135	110	138	137						
				69	80	158	129						
	**C3orf65**												
	(no ortholog)												
**5q13.3**	**HMGCR**	**CG10367**	**V108617**	**0**	**131**	**0**	**563**	**toxic knockdown**					
low-density lipoprotein	(one-to-one ortholog)	HMG Coenzyme A reductase		0	39	0	36						
Kathiresan: rs12654264				0	92	0	450						
						0	77						
	**COL4A3BP**	**CG7207**	**V27914**	**48**	**38**	**52**	**50**	**0.1934**	–0.4214	0.8114	–0.8764	1.2745	**5.5851E–01**
	(one-to-one ortholog)	ceramide transfer protein		14	15	35	38						
				34	23	17	12						
**6p22.3**	**CDKAL1**	**CG6550**	**V4947**	**111**	**101**	**143**	**146**	**0.1149**	–0.2552	0.4857	–0.5360	0.7683	**5.2831E–01**
type 2 diabetes	(one-to-one ortholog)			69	63	10	14						
Scott: rs7754840				42	38	65	70						
						68	62						
**6p21.32**	**HLA-DPA1**												
low-density lipoprotein	(no ortholog)												
Willer: rs2254287													
	**COL11A2**												
	(no ortholog)												
	**RXRB**	**CG4380**	**V16893**	**97**	**190**	**299**	**332**	**–0.5670**	–0.8699	–0.2680	–1.1058	–0.0424	**1.3486E–04**
	(many-to-one ortholog)	ultraspiracle		40	72	66	63						
				33	63	115	113						
				24	55	118	156						
	**SLC39A7**	**CG10449**	**V7183***	**0**	**89**	**0**	**184**	**toxic knockdown**					
	(one-to-one ortholog)	Catecholamines up		0	50	0	29						
				0	19	0	82						
				0	20	0	73						

continued on next page

				scoring: tub$_{P}$ > RNAi					confidence interval bounds				
				1.0 M sucrose		0.15 M sucrose			95%		Bonferroni		
**Locus**	**Human Gene**	**Fly Gene**	**Insertion**	RNAi	control	RNAi	control	**ln(OR)**	lower	upper	lower	upper	**p-value**
	**HSD17B8**	**CG3603**	**V51662***	**48**	**24**	**44**	**34**	**0.4324**	–0.2793	1.1547	–0.7977	1.6992	**2.4075E–01**
	(one-to-one ortholog)			13	8	15	13						
				18	10	7	7						
				17	6	22	14						
	**MIR219-1**												
	(no ortholog)												
	**RING1**	**CG5595**	**V27465**	**29**	**451**	**342**	**568**	**–2.2355**	–2.6711	–1.8321	–3.0367	–1.5530	**1.1701E–42**
	(many-to-one ortholog)	Sex combs extra		9	43	45	61						
				5	96	23	115						
				4	59	48	94						
				0	59	39	74						
				6	57	52	58						
				2	36	66	93						
				3	101	69	73						
			**V106328**	**0**	**520**	**0**	**484**	**toxic knockdown**					
				0	189	0	111						
				0	148	0	173						
				0	183	0	200						
	**VPS52**	**CG7371**	**V27985**	**64**	**58**	**56**	**63**	**0.2153**	–0.3207	0.7535	–0.7204	1.1586	**4.4048E–01**
	(one-to-one ortholog)			30	31	44	40						
				34	27	12	23						
			**V48711**	**145**	**162**	**258**	**322**	**0.1106**	–0.1768	0.3978	–0.3974	0.6178	**4.3665E–01**
				79	84	105	151						
				66	78	153	171						
	**RPS18**	**CG8900**	**V23083**	**285**	**293**	**298**	**274**	**–0.1116**	–0.3497	0.1264	–0.5335	0.3096	**3.4608E–01**
	(many-to-one ortholog)	Ribosomal protein S18		88	85	53	51						
				40	40	30	41						
				157	168	215	182						
**7p15.2**	**JAZF1**	**CG12054**	**V45983**	**125**	**138**	**229**	**247**	**–0.0232**	–0.3364	0.2895	–0.5764	0.5284	**9.3871E–01**
type 2 diabetes	(one-to-one ortholog)			55	69	79	86						
Zeggini: rs864745				70	69	150	161						
			**V45986**	**174**	**153**	**289**	**257**	**0.0113**	–0.2727	0.2956	–0.4903	0.5141	**9.4426E–01**
				63	51	104	78						
				111	102	185	179						
**7p13**	**AEBP1**												
fasting glucose	(no ortholog)												
Dupuis: rs4607517													
	**MIR4649**												
	(no ortholog)												
	**POLD2**	**CG12018**	not tested										
	(one-to-one ortholog)	BcDNA:HL03874											
	**MYL7**	**CG2184**	not tested										
	(many-to-one ortholog)												

continued on next page

				scoring: tub$_P$ > RNAi					confidence interval bounds				
				1.0 M sucrose		0.15 M sucrose			95%		Bonferroni		
Locus	Human Gene	Fly Gene	Insertion	RNAi	control	RNAi	control	ln(OR)	lower	upper	lower	upper	p-value
	**GCK**	**CG3001**	**V21054**	**0**	**420**	**315**	**349**	**−∞**	−∞	−4.6121	−∞	−3.7697	**7.9623E−85**
	(many-to-many ortholog)	Hexokinase A		0	88	51	61						
				0	137	128	114						
				0	195	136	174						
			**V104680**	**280**	**333**	**405**	**474**	**−0.0160**	−0.2285	0.1963	−0.3930	0.3602	**9.1591E−01**
				89	42	137	150						
				109	109	148	202						
				82	182	120	122						
		**CG8094**	**V35337**	**208**	**368**	**344**	**445**	**−0.3129**	−0.5403	−0.0865	−0.7168	0.0872	**6.1959E−03**
		Hexokinase C		102	186	196	229						
				76	100	55	65						
				30	82	93	151						
			**V35338**	**12**	**561**	**161**	**466**	**−2.7803**	−3.4739	−2.1786	−4.0913	−1.7942	**4.6097E−36**
				1	222	92	259						
				9	221	37	107						
				2	118	32	100						
		**CG32849**	**V47331**	**355**	**431**	**336**	**379**	**−0.0735**	−0.2820	0.1349	−0.4432	0.2960	**5.0040E−01**
		Hex-t2		63	76	93	81						
				229	288	53	51						
				63	67	190	247						
			**V100218**	**120**	**212**	**220**	**168**	**−0.8376**	−1.1511	−0.5269	−1.3937	−0.2921	**4.1872E−08**
				58	108	38	27						
				54	96	96	98						
				8	8	86	43						
		**CG33102**	**V46573**	**220**	**246**	**285**	**321**	**0.0072**	−0.2421	0.2565	−0.4341	0.4482	**1.0000E+00**
		Hex-t1		59	52	73	96						
				119	129	73	74						
				42	65	139	151						
			**V46574**	**323**	**344**	**687**	**756**	**0.0327**	−0.1553	0.2206	−0.3012	0.3662	**7.4297E−01**
				159	177	195	213						
				102	87	266	260						
				62	80	226	283						
	**YKT6**	**CG1515**	not tested										
	(one-to-one ortholog)												
	**CAMK2B**												
	(no ortholog)												
**7q11.23**	**BAZ1B**	**CG4254**	no RNAi										
triglyceride	(one-to-many ortholog)	twinstar											
Willer: rs17145738													
Kooner: rs3812316		**CG6873**	**V22454**	**0**	**28**	**1**	**226**	**toxic knockdown**					
				~~75~~	~~121~~	0	23						
				0	28	0	24						
						0	11						
						1	83						
						0	85						

continued on next page

Locus	Human Gene	Fly Gene	Insertion	scoring: $tub_P > RNAi$				ln(OR)	confidence interval bounds				p-value
				1.0 M sucrose		0.15 M sucrose			95%		Bonferroni		
				RNAi	control	RNAi	control		lower	upper	lower	upper	
	**BCL7B**	**CG17252**	**V20410**	**303**	**309**	**225**	**217**	**–0.0557**	–0.3081	0.1965	–0.5025	0.3905	**6.6260E–01**
	(many-to-one ortholog)	BCL7-like		108	108	78	53						
				112	115	89	110						
				83	86	58	54						
			**V106828**	**333**	**419**	**278**	**309**	**–0.1239**	–0.3466	0.0987	–0.5186	0.2704	**2.6919E–01**
				117	154	71	71						
				96	129	81	98						
				120	136	126	140						
	**TBL2**												
	(no ortholog)												
	**MLXIPL**	**CG18362**	**V52606**	**128**	**230**	**264**	**263**	**–0.5892**	–0.8754	–0.3053	–1.0972	–0.0898	**2.5790E–05**
	(many-to-one ortholog)	Mio		54	112	104	99						
				50	86	105	105						
				24	32	55	59						
			**V52607**	**166**	**288**	**390**	**400**	**–0.5252**	–0.7697	–0.2825	–0.9598	–0.0972	**1.4864E–05**
				59	87	121	125						
				100	183	133	163						
				7	18	136	112						
	**VPS37D**	**CG1115**	not tested										
	(many-to-one ortholog)												
	**DNAJC30**	**CG11035**	**V8478**	**126**	**106**	**140**	**180**	**0.4234**	0.0699	0.7786	–0.1977	1.0507	**1.5748E–02**
	(one-to-one ortholog)			48	48	14	13						
				78	58	58	59						
						68	108						
	**WBSCR22**	**CG10903**	**V27334**	**0**	**164**	**0**	**321**	**toxic knockdown**					
	(one-to-one ortholog)			0	72	0	92						
				0	92	0	229						
	**STX1A**												
	(no ortholog)												
**8p21.3**	**LPL**	**CG5966**	**V13163**	**45**	**61**	**35**	**35**	**–0.3025**	–0.9551	0.3473	–1.4428	0.8281	**3.5573E–01**
triglyceride,	(many-to-many ortholog)	(shared: LIPC (15q21.3)		2	8	35	35						
high-density lipoprotein		and LIPG (18q21.1))		20	18								
Willer: rs10503669				23	35								
Kathiresan: rs328													
			**V13164**	**181**	**203**	**211**	**213**	**–0.1051**	–0.3913	0.1807	–0.6110	0.3995	**4.8116E–01**
				55	64	53	59						
				40	30	35	33						
				86	109	123	121						
		**CG6847**	**V22451**	**0**	**87**	**21**	**72**	**$-\infty$**	$-\infty$	–1.7861	$-\infty$	–0.8024	**3.3388E–07**
		(shared: LIPC (15q21.3)		0	29	6	25						
		and LIPG (18q21.1))		0	21	5	13						
				0	37	10	34						

continued on next page

				scoring: tub$_P$ > RNAi					confidence interval bounds				
				1.0 M sucrose		0.15 M sucrose			95%		Bonferroni		
**Locus**	**Human Gene**	**Fly Gene**	**Insertion**	RNAi	control	RNAi	control	**ln(OR)**	lower	upper	lower	upper	**p-value**
**8q24.11**	**SLC30A8**	**CG3994**	**V3836**	**76**	**74**	**56**	**80**	**0.3820**	–0.1113	0.8788	–0.4790	1.2555	**1.2304E–01**
type 2 diabetes	(many-to-many ortholog)	ZnT35C		22	21	18	23						
Scott: rs13266634				27	24	11	9						
				27	29	27	48						
			**V103263**	**691**	**636**	**509**	**570**	**0.1960**	0.0320	0.3603	–0.0954	0.4881	**1.7454E–02**
				182	165	101	99						
				292	296	216	203						
				217	175	192	268						
		**CG31860**	**V7688**	**0**	**375**	**0**	**403**	**toxic knockdown**					
				0	36	0	61						
				0	112	0	179						
				0	227	0	163						
			**V103398**	**422**	**772**	**417**	**570**	**–0.2913**	–0.4685	–0.1143	–0.6062	0.0229	**1.0656E–03**
				104	137	59	68						
				146	244	150	166						
				172	391	208	336						
**8q24.13**	**TRIB1**	**CG5408**	**V22113**	**280**	**347**	**598**	**626**	**–0.1687**	–0.3670	0.0291	–0.5209	0.1818	**9.4502E–02**
triglyceride	(many-to-one ortholog)	tribbles		108	134	160	182						
Willer: rs17321515				115	141	173	182						
				57	72	265	262						
			**V22114**	**210**	**291**	**458**	**521**	**–0.1972**	–0.4210	0.0258	–0.5947	0.1971	**7.7562E–02**
				118	152	87	85						
				61	88	174	220						
				31	51	197	216						
			**V106774**	**461**	**582**	**498**	**475**	**–0.2802**	–0.4596	–0.1012	–0.5990	0.0375	**1.7888E–03**
				149	185	79	60						
				115	152	124	85						
				181	169	166	178						
				16	76	129	152						
**9p21.3**	**MTAP**	**CG4802**	not tested										
type 2 diabetes	(one-to-many ortholog)												
Scott: rs10811661													
		**CG31115**	not tested										
	**CDKN2A**												
	(no ortholog)												
	**CDKN2B**												
	(no ortholog)												
**9q31.1**	**ABCA1**												
high-density lipoprotein	(no ortholog)												
Willer: rs4149268													
Kathiresan: rs3890182													
**10p13**	**NUDT5**												
type 2 diabetes	(no ortholog)												
Zeggini: rs12779790													
	**CDC123**												
	(no ortholog)												
	**CAMK1D**												
	(no ortholog)												

continued on next page

				scoring: $tub_P > RNAi$					confidence interval bounds				
				1.0 M sucrose		0.15 M sucrose			95%		Bonferroni		
**Locus**	**Human Gene**	**Fly Gene**	**Insertion**	RNAi	control	RNAi	control	**ln(OR)**	lower	upper	lower	upper	**p-value**
**10q23.33**	**IDE**	**CG5517**	**V15957**	**55**	**178**	**66**	**216**	**0.0112**	–0.4204	0.4403	–0.7504	0.7639	**1.0000E+00**
type 2 diabetes	(one-to-one ortholog)	Insulin degrading		38	98	6	17						
Scott: rs1111875		metalloproteinase		17	80	17	101						
						43	98						
			**V15958**	**71**	**174**	**252**	**411**	**–0.4068**	–0.7404	–0.0797	–1.0023	0.1644	**1.2433E–02**
				29	54	9	12						
				3	39	26	54						
				20	21	72	85						
				9	16	45	71						
				10	44	62	94						
						38	95						
			**V101317**	**191**	**185**	**170**	**183**	**0.1055**	–0.1956	0.4069	–0.4258	0.6380	**5.0496E–01**
				82	92	120	126						
				38	43	23	24						
				71	50	27	33						
	**KIF11**	**CG9191**	**V52548**	**166**	**223**	**332**	**363**	**–0.2057**	–0.4642	0.0518	–0.6639	0.2489	**1.1241E–01**
	(one-to-one ortholog)	Kinesin-like protein at 61F		62	80	112	84						
				46	94	129	153						
				32	14	14	15						
				13	14	27	32						
				13	21	50	79						
			**V52549**	**113**	**94**	**243**	**331**	**0.4925**	0.1606	0.8262	–0.0914	1.0830	**2.6050E–03**
				76	57	112	143						
				28	28	121	170						
				9	9	10	18						
			**V109280**	**163**	**217**	**179**	**194**	**–0.2054**	–0.5034	0.0919	–0.7322	0.3191	**1.6503E–01**
				61	97	131	133						
				49	42	18	32						
				53	78	30	29						
	**HHEX**	**CG7056**	**V15719**	**0**	**59**	**46**	**72**	**–∞**	–∞	–2.2184	–∞	–1.2822	**2.2235E–10**
	(one-to-one ortholog)			0	14	4	9						
				0	16	6	23						
				~~0~~	~~4~~	36	40						
				0	9								
				0	20								
			**V15721**	**9**	**263**	**647**	**725**	**–3.2599**	–4.0624	–2.5923	–4.8014	–2.1858	**2.1611E–52**
				7	106	19	12						
				1	70	53	84						
				1	87	108	103						
						68	90						
						356	383						
						43	53						
			**V100292**	**0**	**354**	**13**	**285**	**–∞**	–∞	–1.3184	–∞	–0.2890	**3.2854E–05**
				0	184	2	142						
				0	77	4	70						
				0	93	7	73						

continued on next page

				scoring: tub$_P$ > RNAi					confidence interval bounds				
				1.0 M sucrose		0.15 M sucrose			95%		Bonferroni		
**Locus**	**Human Gene**	**Fly Gene**	**Insertion**	RNAi	control	RNAi	control	**ln(OR)**	lower	upper	lower	upper	**p-value**
**10q25.2**	**TCF7L2**	**CG34403**	**V3014**	**7**	**32**	**82**	**77**	**–1.5754**	–2.6214	–0.6662	–3.5391	–0.0785	**1.3333E–04**
type 2 diabetes	(many-to-one ortholog)	pangolin		7	32	71	53						
Sladek: rs7903146						11	24						
**11p15.1**	**NUCB2**	**CG32190**	**V5319**	**224**	**212**	**543**	**546**	**0.0605**	–0.1679	0.2891	–0.3440	0.4658	**6.1022E–01**
type 2 diabetes	(many-to-one ortholog)	NUCB1		101	88	105	95						
Scott: rs5219				92	101	194	215						
				31	23	244	236						
			**V100688**	**230**	**330**	**290**	**289**	**–0.3641**	–0.6057	–0.1233	–0.7926	0.0615	**2.4160E–03**
				74	133	80	73						
				79	91	139	145						
				77	106	71	71						
	**NCR3LG1**												
	(no ortholog)												
	**KCNJ11**	**CG4370**	**V4341**	**84**	**260**	**21**	**226**	**1.2442**	0.7187	1.8075	0.3522	2.2708	**3.6149E–07**
	(many-to-many ortholog)	Inwardly rectifying		33	138	9	84						
		potassium channel 2		9	34	12	101						
				42	88	0	41						
			**V108140**	**0**	**72**	**0**	**304**	**toxic knockdown**					
				0	14	0	103						
				0	35	0	117						
				0	23	0	84						
		**CG6747**	**V28430**	**0**	**338**	**0**	**482**	**toxic knockdown**					
		Inwardly rectifying		0	135	0	219						
		potassium channel		0	73	0	123						
				0	130	0	140						
			**V28431**	**0**	**482**	**0**	**484**	**toxic knockdown**					
				0	55	0	254						
				0	165	0	79						
				0	262	0	151						
			**V107389**	**81**	**115**	**195**	**193**	**–0.3602**	–0.7238	0.0007	–1.0035	0.2729	**4.3830E–02**
				18	24	82	68						
				30	46	57	64						
				33	45	56	61						
		**CG10369**	**V3886**	**232**	**216**	**187**	**222**	**0.2427**	–0.0347	0.5208	–0.2470	0.7349	**8.7180E–02**
		Inwardly rectifying		131	94	45	52						
		potassium channel 3		43	56	102	118						
				58	66	40	52						
			**V101174**	**103**	**282**	**288**	**387**	**–0.7111**	–0.9950	–0.4314	–1.2174	–0.2207	**2.2441E–07**
				56	160	49	54						
				21	48	167	250						
				26	74	72	83						
	**ABCC8**	**CG7806**	**V2804**	**265**	**216**	**626**	**639**	**0.2249**	0.0087	0.4416	–0.1576	0.6099	**3.6819E–02**
	(many-to-one ortholog)			123	101	175	201						
				82	76	252	241						
				60	39	199	197						

continued on next page

				scoring: tub_P > RNAi					confidence interval bounds				
				1.0 M sucrose		0.15 M sucrose			95%		Bonferroni		
Locus	Human Gene	Fly Gene	Insertion	RNAi	control	RNAi	control	ln(OR)	lower	upper	lower	upper	p-value
**11p11.2**	**EXT2**	**CG8433**	**V4902**	**139**	**153**	**175**	**173**	**–0.1073**	–0.4310	0.2159	–0.6786	0.4624	**5.2576E–01**
type 2 diabetes	(one-to-one ortholog)	Ext2		94	103	14	14						
Sladek: rs1113132				45	50	80	83						
rs11037909						81	76						
rs3740878													
			**V4903**	**50**	**43**	**215**	**196**	**0.0582**	–0.4175	0.5370	–0.7755	0.9028	**8.1903E–01**
				22	21	5	16						
				28	22	81	71						
						129	109						
			**V49808***	**4**	**63**	**3**	**101**	**toxic knockdown**					
				3	46	3	66						
				0	8	0	35						
				1	9								
			**V49809**	**85**	**86**	**254**	**234**	**–0.0936**	–0.4575	0.2702	–0.7352	0.5473	**6.5675E–01**
				34	31	143	113						
				51	55	111	121						
	**ALX4**												
	(no ortholog)												
**11q23.3**	**BUD13**	**CG13625**	not tested										
triglyceride	(one-to-one ortholog)												
Willer: rs12286037													
Kathiresan: rs28927680	**ZNF259**	**CG9060**	**V12665***	**0**	**93**	**1**	**191**	**toxic knockdown**					
	(one-to-one ortholog)	Zpr1		0	32	1	47						
				0	37	0	86						
				0	24	0	58						
	**APOA5**												
	(no ortholog)												
	**APOA4**												
	(no ortholog)												
	**APOC3**												
	(no ortholog)												
	**APOA1**												
	(no ortholog)												
	**SIK3**	**CG42856**	not tested										
	(one-to-one ortholog)	Salt-inducible kinase 3											
**12q15**	**TSPAN8**	**CG6120**	**V3422**	**179**	**169**	**163**	**195**	**0.2364**	–0.0700	0.5435	–0.3037	0.7791	**1.3196E–01**
type 2 diabetes	(many-to-one ortholog)	Tetraspanin 96F		49	52	33	42						
Zeggini: rs7961581				42	27	111	122						
				88	90	19	31						
			**V3423**	**190**	**200**	**155**	**155**	**–0.0512**	–0.3608	0.2582	–0.5977	0.4948	**7.6107E–01**
				33	21	47	42						
				44	43	69	72						
				113	136	39	41						
			**V101411**	**208**	**264**	**179**	**227**	**–0.0008**	–0.2770	0.2755	–0.4888	0.4877	**1.0000E+00**
				72	105	52	69						
				76	93	81	100						
				60	66	46	58						
	**LGR5**												
	(no ortholog)												

continued on next page

				scoring: tub_P > RNAi					confidence interval bounds				
				1.0 M sucrose		0.15 M sucrose			95%		Bonferroni		
**Locus**	**Human Gene**	**Fly Gene**	**Insertion**	RNAi	control	RNAi	control	**ln(OR)**	lower	upper	lower	upper	**p-value**
**12q23.3**	**MYO1H**												
high-density lipoprotein	(no ortholog)												
Willer: rs2338104													
	**KCTD10**	**CG10465**	**V107131**	**198**	**313**	**244**	**255**	**–0.4134**	–0.6718	–0.1560	–0.8713	0.0410	**1.2206E–03**
	(many-to-one ortholog)			75	103	46	34						
				54	99	145	170						
				69	111	53	51						
	**UBE3B**	**CG5087**	not tested										
	(one-to-one ortholog)												
	**MMAB**												
	(no ortholog)												
	**MVK**	**CG33671**	**V49772***	**10**	**65**	**34**	**39**	**–1.7222**	–2.6514	–0.8691	–3.4127	–0.2930	**1.1478E–05**
	(one-to-one ortholog)			2	21	20	26						
				6	23	14	13						
				2	21								
**15q21.3**	**LIPC**	**CG5966**	**V13163**	**45**	**61**	**35**	**35**	**–0.3025**	–0.9551	0.3473	–1.4428	0.8281	**3.5573E–01**
high-density lipoprotein	(many-to-many ortholog)	(shared: LPL (8p21.3)		2	8	35	35						
Willer: rs4775041		and LIPG (18q21.1))		20	18								
Kathiresan: rs1800588				23	35								
			**V13164**	**181**	**203**	**211**	**213**	**–0.1051**	–0.3913	0.1807	–0.6110	0.3995	**4.8116E–01**
				55	64	53	59						
				40	30	35	33						
				86	109	123	121						
		**CG6847**	**V22451**	**0**	**87**	**21**	**72**	$-\infty$	$-\infty$	–1.7861	$-\infty$	–0.8024	**3.3388E–07**
		(shared: LPL (8p21.3)		0	29	6	25						
		and LIPG (18q21.1))		0	21	5	13						
				0	37	10	34						
**16q12.1**	**RPGRIP1L**												
type 2 diabetes	(no ortholog)												
Scott: rs8050136													
	**FTO**												
	(no ortholog)												
**16q12.2**	**CETP**												
high-density lipoprotein	(no ortholog)												
Willer: rs9989419													
rs3764261	**NLRC5**												
rs1864163	(no ortholog)												
Kathiresan: rs1800775													

continued on next page

				scoring: tub$_P$ > RNAi					confidence interval bounds				
				1.0 M sucrose		0.15 M sucrose			95%		Bonferroni		
**Locus**	**Human Gene**	**Fly Gene**	**Insertion**	RNAi	control	RNAi	control	**ln(OR)**	lower	upper	lower	upper	**p-value**
	**SLC12A3**	**CG4357**	**V30000**	**473**	**445**	**269**	**381**	**0.4088**	0.2012	0.6172	0.0414	0.7790	**7.7131E–05**
	(many-to-many ortholog)	sodium chloride		214	203	42	58						
		cotransporter 69		138	136	100	92						
				121	106	127	231						
			**V30001**	**232**	**244**	**539**	**547**	**–0.0357**	–0.2572	0.1857	–0.4284	0.3566	**7.8344E–01**
				75	59	190	198						
				2	1	151	143						
				155	184	198	206						
			**V106499**	**465**	**535**	**363**	**376**	**–0.1050**	–0.3000	0.0900	–0.4511	0.2408	**2.8562E–01**
				131	168	82	84						
				182	183	198	182						
				152	184	83	110						
		**CG31547**	**V8551**	**337**	**420**	**273**	**299**	**–0.1291**	–0.3530	0.0948	–0.5259	0.2674	**2.6624E–01**
				44	54	74	64						
				113	135	100	101						
				180	231	99	134						
			**V8552**	**96**	**118**	**83**	**72**	**–0.3475**	–0.7850	0.0877	–1.1176	0.4147	**1.1362E–01**
				24	42	18	19						
				6	20	5	8						
				66	56	60	45						
			**V105911**	**426**	**511**	**441**	**426**	**–0.2164**	–0.4060	–0.0271	–0.5531	0.1194	**2.3589E–02**
				121	148	64	63						
				148	187	212	223						
				157	176	165	140						
	**HERPUD1**	**CG14536**	**V11724**	**198**	**167**	**614**	**539**	**0.0400**	–0.2030	0.2837	–0.3897	0.4722	**7.6354E–01**
	(many-to-one ortholog)	Homocysteine-induced		94	64	110	98						
		endoplasmic reticulum protein		56	45	299	252						
				48	58	205	189						
			**V11725**	**56**	**51**	**147**	**152**	**0.1267**	–0.3391	0.5940	–0.6900	0.9490	**6.5239E–01**
				11	8	13	14						
				8	11	49	50						
				37	32	85	88						
	**MIR138-2**												
	(no ortholog)												
**18q21.1**	**LIPG**	**CG5966**	**V13163**	**45**	**61**	**35**	**35**	**–0.3025**	–0.9551	0.3473	–1.4428	0.8281	**3.5573E–01**
high-density lipoprotein	(many-to-many ortholog)	(shared: LPL (8p21.3)		2	8	35	35						
Willer: rs2156552		and LIPC (15q21.3))		20	18								
				23	35								
			**V13164**	**181**	**203**	**211**	**213**	**–0.1051**	–0.3913	0.1807	–0.6110	0.3995	**4.8116E–01**
				55	64	53	59						
				40	30	35	33						
				86	109	123	121						
		**CG6847**	**V22451**	**0**	**87**	**21**	**72**	**$-\infty$**	$-\infty$	–1.7861	$-\infty$	–0.8024	**3.3388E–07**
		(shared: LPL (8p21.3)		0	29	6	25						
		and LIPC (15q21.3))		0	21	5	13						
				0	37	10	34						
	**ACAA2**	**CG4600**	**V26562**	**92**	**62**	**216**	**191**	**0.2712**	–0.1209	0.6675	–0.4153	0.9730	**1.8314E–01**
	(one-to-one ortholog)	yippee interacting protein 2		25	18	103	102						
				67	44	113	89						

continued on next page

				scoring: $tub_P > RNAi$					confidence interval bounds				
				1.0 M sucrose		0.15 M sucrose			95%		Bonferroni		
**Locus**	**Human Gene**	**Fly Gene**	**Insertion**	RNAi	control	RNAi	control	**ln(OR)**	lower	upper	lower	upper	**p-value**
**19p13.2**	**SMARCA4**	**CG5942**	**V37720**	**0**	**197**	**0**	**189**	**toxic knockdown**					
low-density lipoprotein	(many-to-one ortholog)	brahma		0	62	0	36						
Willer: rs6511720				0	70	0	76						
				0	25	0	46						
				0	40	0	31						
			**V37721**	**0**	**266**	**1**	**315**	**toxic knockdown**					
				0	77	0	64						
				0	62	0	64						
				0	100	0	65						
				0	27	1	122						
	**LDLR**												
	(no ortholog)												
	**SPC24**												
	(no ortholog)												
	**KANK2**												
	(no ortholog)												
**19p13.11**	**GATAD2A**	**CG32067**	**V13066**	**281**	**500**	**271**	**539**	**0.1113**	–0.1006	0.3233	–0.2643	0.4873	**2.9266E–01**
triglyceride,	(many-to-one ortholog)	simjang		71	174	52	107						
low-density lipoprotein				115	172	80	167						
Willer: rs16996148				95	154	139	265						
			**V100285**	**0**	**437**	**0**	**303**	**toxic knockdown**					
				0	67	0	64						
				0	108	0	112						
				0	262	0	127						
	**TSSK6**	**CG14305**	**V17477**	**388**	**356**	**485**	**512**	**0.1402**	–0.0544	0.3349	–0.2048	0.4859	**1.6002E–01**
	(many-to-one ortholog)			161	137	78	77						
				126	110	156	157						
				101	109	251	278						
	**NDUFA13**	**CG3446**	**V42696***	**1**	**120**	**174**	**174**	**–4.7813**	–8.4498	–3.0197	$-\infty$	–2.3002	**4.1032E–28**
	(one-to-one ortholog)			1	80	37	54						
				0	24	68	57						
				0	16	69	63						
	**YJEFN3**	**CG2974**	not tested										
	(many-to-one ortholog)												
	**CILP2**												
	(no ortholog)												

continued on next page

				scoring: $tub_P > RNAi$					confidence interval bounds				
				1.0 M sucrose		0.15 M sucrose			95%		Bonferroni		
**Locus**	**Human Gene**	**Fly Gene**	**Insertion**	RNAi	control	RNAi	control	**ln(OR)**	lower	upper	lower	upper	**p-value**
	**PBX4**	**CG8933**	**V7802**	**0**	**164**	**0**	**66**	**toxic knockdown**					
	(many-to-one ortholog)	extradenticle		0	19	0	47						
				0	56	0	7						
				0	89	0	12						
			**V7803**	**0**	**106**	**0**	**268**	**toxic knockdown**					
				0	35	0	57						
				0	52	0	113						
				~~5~~	~~41~~	0	98						
				0	19								
			**V100259**	**3**	**379**	**0**	**315**	**toxic knockdown**					
				3	115	0	49						
				0	104	0	82						
				0	160	0	184						
			**V100687**	**17**	**315**	**11**	**187**	**–0.0860**	–0.9274	0.7963	–1.5376	1.5059	**8.4274E–01**
				2	67	1	37						
				8	86	1	49						
				7	162	9	101						
	**LPAR2**												
	(no ortholog)												
	**GMIP**												
	(no ortholog)												
	**ATP13A1**	**CG6230**	**V8897**	**0**	**134**	**44**	**179**	**$-\infty$**	$-\infty$	–2.1193	$-\infty$	–1.2139	**3.4181E–10**
	(one-to-one ortholog)			0	86	9	14						
				0	48	21	74						
						14	91						
**19q13.33**	**BCL3**												
low-density lipoprotein	(no ortholog)												
Willer: rs10402271													
rs4420638	**CBLC**												
	(no ortholog)												
	**BCAM**												
	(no ortholog)												

continued on next page

Locus	Human Gene	Fly Gene	Insertion	scoring: tub$_P$ > RNAi 1.0 M sucrose RNAi	1.0 M sucrose control	0.15 M sucrose RNAi	0.15 M sucrose control	ln(OR)	confidence interval bounds 95% lower	95% upper	Bonferroni lower	Bonferroni upper	p-value
	**PVRL2**	**CG17716**	**V6561**	**271**	**297**	**362**	**395**	**–0.0044**	–0.2282	0.2194	–0.4012	0.3921	**1.0000E+00**
	(many-to-one ortholog)	faint sausage		92	126	130	126						
				109	108	121	140						
				70	63	111	129						
			**V6562**	**131**	**138**	**116**	**137**	**0.1141**	–0.2444	0.4732	–0.5167	0.7470	**5.3981E–01**
				65	73	70	83						
				47	37	23	28						
				19	28	23	26						
			**V24476**	**359**	**359**	**640**	**649**	**0.0140**	–0.1728	0.2007	–0.3176	0.3455	**8.8897E–01**
				177	175	84	119						
				52	57	132	125						
				130	127	424	405						
			**V42236**	**177**	**273**	**235**	**317**	**–0.1339**	–0.3958	0.1273	–0.5978	0.3276	**3.0269E–01**
				63	82	56	97						
				80	120	138	146						
				34	71	41	74						
			**V42237**	**103**	**167**	**154**	**200**	**–0.2215**	–0.5587	0.1139	–0.8176	0.3683	**1.8956E–01**
				27	33	27	20						
				47	79	84	103						
				29	55	43	77						
			**V102073**	**125**	**159**	**121**	**146**	**–0.0527**	–0.4029	0.2975	–0.6699	0.5641	**7.9717E–01**
				55	63	29	40						
				33	54	62	72						
				37	42	30	34						
	**TOMM40**	**CG8330**	**V23763**	**0**	**98**	**1**	**38**	**toxic knockdown**					
	(many-to-many ortholog)	tomboy40		0	69	0	32						
				0	4	1	5						
				0	25	0	1						
			**V23764**	**92**	**226**	**86**	**235**	**0.1063**	–0.2546	0.4679	–0.5291	0.7442	**5.9658E–01**
				45	91	14	50						
				8	31	44	118						
				39	104	28	67						
			**V42439**	**2**	**626**	**35**	**882**	**–2.5185**	–4.6722	–1.1531	–7.1161	–0.4745	**1.5529E–06**
				2	182	4	98						
				0	22	1	175						
				0	176	12	294						
				0	95	1	61						
				0	77	3	103						
				0	74	14	151						
			**V105557**	**1**	**427**	**4**	**615**	**toxic knockdown**					
				0	207	0	47						
				1	139	4	308						
				0	81	0	260						
		**CG12157**	**V13177**	**0**	**309**	**0**	**303**	**toxic knockdown**					
		Translocase of		0	130	0	42						
		outer membrane 40		0	113	0	108						
				0	66	0	153						
			**V13178**	**135**	**318**	**156**	**366**	**–0.0040**	–0.2890	0.2804	–0.5083	0.4980	**1.0000E+00**
				59	172	76	207						
				41	68	47	81						
				35	78	33	78						

continued on next page

Locus	Human Gene	Fly Gene	Insertion	scoring: tub_P > RNAi 1.0 M sucrose RNAi	scoring: tub_P > RNAi 1.0 M sucrose control	scoring: tub_P > RNAi 0.15 M sucrose RNAi	scoring: tub_P > RNAi 0.15 M sucrose control	ln(OR)	confidence interval bounds 95% lower	confidence interval bounds 95% upper	confidence interval bounds Bonferroni lower	confidence interval bounds Bonferroni upper	p-value
	**APOE**												
	(no ortholog)												
	**APOC1**												
	(no ortholog)												
	**APOC4**												
	(no ortholog)												
	**APOC2**												
	(no ortholog)												
	**CLPTM1**	**CG3702**	not tested										
	(one-to-one ortholog)												
	**RELB**	**CG6667**	**V45996**	**370**	**559**	**293**	**303**	**–0.3788**	–0.5919	–0.1660	–0.7569	–0.0019	**3.8680E–04**
	(many-to-one ortholog)	dorsal		96	130	53	52						
				91	196	35	52						
				111	152	87	75						
				72	81	118	124						
			**V45998**	**183**	**216**	**117**	**136**	**–0.0153**	–0.3436	0.3135	–0.5939	0.5651	**9.3589E–01**
				51	72	29	32						
				56	75	36	31						
				40	42	36	38						
				36	27	16	35						
			**V105491**	**19**	**640**	**566**	**784**	**–3.1901**	–3.7162	–2.7203	–4.1735	–2.4106	**8.1235E–92**
				1	140	31	56						
				0	102	52	78						
				2	150	14	73						
				4	56	141	132						
				3	52	85	100						
				9	58	121	127						
				0	82	122	218						

Table S1. RNAi screen for sucrose sensitivity: knockdowns. Experimental data associated with Figures 1–2. Human genomic regions and SNPs, human genes within ~100 kb radius window and their Ensembl 43 fly orthologs, fly RNAi lines (as VDRC catalog numbers), and experimental results are shown, as well as p-values and confidence intervals for the null hypothesis that the odds of knockdown vs. non-knockdown survival are the same on 1.0 M sucrose as on 0.15 M sucrose. Survival to pupariation was scored except for a small number of crosses, marked in the table with asterisks, where survival to eclosion was scored for technical reasons. In the "scoring" columns, bold numbers are total counts, and counts for individual trials are listed immediately underneath them.

Table S2 – RNAi backgrounds
(data associated with Figure 1)

	scoring: $tub_P > RNAi$					confidence interval bounds				
	1.0 M sucrose		0.15 M sucrose			95%		Bonferroni		
**Fly Strain**	RNAi	control	RNAi	control	**ln(OR)**	lower	upper	lower	upper	**p-value**
**GD control: w^{1118}**	**261**	**278**	**1497**	**1526**	**–0.0439**	–0.2317	0.1437	–0.3776	0.2891	**6.4045E–01**
	39	39	295	318						
	71	64	382	396						
	41	49	76	74						
	9	13	78	80						
	12	14	71	69						
	70	76	105	73						
	19	23	95	110						
			194	159						
			109	119						
			92	128						
**KK control: $y^- w^{1118}$; P{attP y^+ $w^{3'}$}VIE-260B**	**234**	**239**	**419**	**403**	**–0.0600**	–0.2928	0.1727	–0.4725	0.3521	**6.0406E–01**
	89	79	148	146						
	23	31	58	62						
	33	44	106	87						
	34	36	4	11						
	55	49	103	97						

Table S2. RNAi screen for sucrose sensitivity: controls. Control data associated with Figure 1. Survival to pupariation of progeny of w^{1118} and y^- w^{1118}; P{attP, y^+, $w^{3'}$}VIE-260B males crossed to tub_P-GAL4/TM6B unmated females. In the "scoring" columns, bold numbers are total counts, and counts for individual trials are listed immediately underneath them. Similar survival indicates that the genetic backgrounds in the study are not themselves differentially sucrose-sensitive.

Table S3 – diet survey

(data associated with Figure 3A)

	scoring					confidence interval bounds				
	> RNAi$^{dHHEX\text{-}V15721}$		w^{1118} genetic background			95%		Bonferroni		
**Food**	with tub$_P$ >	control	with tub$_P$ >	control	**ln(OR)**	lower	upper	lower	upper	**p-value**
0.15 M sucrose	**647**	**725**	**1497**	**1526**	**–0.0946**	–0.2245	0.0352	–0.2836	0.0941	**1.5196E–01**
	19	12	295	318						
	53	84	382	396						
	108	103	76	74						
	68	90	78	80						
	356	383	71	69						
	43	53	105	73						
			95	110						
			194	159						
			109	119						
			92	128						
0.5 M sucrose	**42**	**83**	**83**	**78**	**–0.7407**	–1.2567	–0.2332	–1.4862	–0.0151	**2.6870E–03**
	42	83	83	78						
0.75 M sucrose	**3**	**10**	**24**	**21**	**–1.3152**	–3.1725	0.2066	–4.1229	0.7668	**6.5918E–02**
	3	10	24	21						
1.0 M sucrose	**9**	**263**	**261**	**278**	**–3.3087**	–4.1234	–2.6238	–4.5385	–2.3728	**3.7116E–46**
	7	106	39	39						
	1	70	71	64						
	1	87	41	49						
			9	13						
			12	14						
			70	76						
			19	23						
0.15 M sucrose + 1.7 M glucose	**0**	**62**	**69**	**75**	**$-\infty$**	$-\infty$	–2.6482	$-\infty$	–2.0831	**3.6364E–14**
	0	62	69	75						
0.15 M sucrose + 1.7 M fructose	**0**	**119**	**108**	**98**	**$-\infty$**	$-\infty$	–3.5113	$-\infty$	–2.9636	**2.2245E–28**
	0	119	108	98						
0.15 M sucrose + 0.1 M glucosamine	**0**	**100**	**27**	**55**	**$-\infty$**	$-\infty$	–2.4568	$-\infty$	–1.8684	**2.7095E–11**
	0	100	27	55						

continued on next page

Food	scoring: > RNAi$^{dHHEX-V15721}$ with tub$_P$>	control	w^{1118} genetic background with tub$_P$>	control	ln(OR)	confidence interval bounds: 95% lower	95% upper	Bonferroni lower	Bonferroni upper	p-value
0.15 M sucrose + 140 mg/mL fat	**226**	**235**	**360**	**416**	**0.1054**	–0.1321	0.3430	–0.2387	0.4497	**3.7758E–01**
	100	107	115	131						
	56	66	123	146						
	70	62	122	139						
0.15 M sucrose + 0.5 M NaCl	**0**	**123**	**77**	**111**	**$-\infty$**	$-\infty$	–3.0785	$-\infty$	–2.5281	**5.6924E–21**
	0	34	23	25						
	0	31	26	39						
	0	58	28	47						
0.15 M sucrose + 10 mM H_2O_2	**197**	**285**	**258**	**296**	**–0.2317**	–0.4864	0.0224	–0.6009	0.1359	**6.8937E–02**
	35	64	57	54						
	79	102	57	52						
	83	119	144	190						
0.15 M sucrose + 600 μM $AgNO_3$	**30**	**89**	**46**	**63**	**–0.7695**	–1.3759	–0.1744	–1.6444	0.0792	**7.6202E–03**
	4	24	9	11						
	6	21	22	30						
	20	44	15	22						
0.15 M sucrose, heat-shocked	**48**	**69**	**65**	**75**	**–0.2189**	–0.7468	0.3058	–0.9784	0.5334	**4.4913E–01**
	17	22	27	26						
	12	27	19	28						
	19	20	19	21						

Table S3. Diet survey. Ubiquitous dHHEX knockdown results in intolerance to sugar and salt but not other stressors. Data associated with Figure 3A: survival to pupariation of progeny of UAS-RNAi$^{dHHEX-V15721}$ and w^{1118} (genetic background control) males crossed to tub$_P$-GAL4/TM6B unmated females, reared on different diets. In the "scoring" columns, bold numbers are total counts, and counts for individual trials are listed immediately underneath them.

Table S4 – driver survey, part 1
(data associated with Figure 3B)

			scoring					confidence interval bounds				
			1.0 M sucrose		0.15 M sucrose			95%		Bonferroni		
**Driver**	**crossed to**	**stage**	RNAi	control	RNAi	control	**ln(OR)**	lower	upper	lower	upper	**p-value**
tub_P-GAL4	w^{1118}	(pupae)	**261**	**278**	**1497**	**1526**	**–0.0439**	–0.2317	0.1437	–0.3140	0.2257	**6.4045E–01**
			39	39	295	318						
			71	64	382	396						
			41	49	76	74						
			9	13	78	80						
			12	14	71	69						
			70	76	105	73						
			19	23	95	110						
					194	159						
					109	119						
					92	128						
	UAS-$RNAi^{dHHEX\text{-}V15721}$	(pupae)	**9**	**263**	**647**	**725**	**–3.2599**	–4.0624	–2.5923	–4.4615	–2.3568	**2.1611E–52**
			7	106	19	12						
			1	70	53	84						
			1	87	108	103						
					68	90						
					356	383						
					43	53						
Dilp2-GAL4	w^{1118}	(eclosers)	**649**	**645**	**823**	**765**	**–0.0669**	–0.2165	0.0827	–0.2823	0.1485	**3.8894E–01**
			258	271	347	310						
			219	203	260	267						
			172	171	216	188						
	UAS-$RNAi^{dHHEX\text{-}V15721}$	(eclosers)	**859**	**822**	**652**	**672**	**0.0742**	–0.0725	0.2210	–0.1370	0.2855	**3.2122E–01**
			155	150	116	145						
			410	370	266	294						
			294	302	270	233						
Sns_{GCN}-GAL4	w^{1118}	(pupae)	**17**	**15**	**114**	**98**	**–0.0260**	–0.8384	0.7954	–1.1773	1.1452	**1.0000E+00**
			17	15	68	64						
					46	34						
	UAS-$RNAi^{dHHEX\text{-}V15721}$	(pupae)	**44**	**24**	**215**	**171**	**0.3763**	–0.1873	0.9598	–0.4189	1.2163	**1.8523E–01**
			27	17	21	17						
			17	7	81	53						
					46	36						
					67	65						

continued on next page

Driver	crossed to	stage	scoring: 1.0 M sucrose RNAi	1.0 M sucrose control	0.15 M sucrose RNAi	0.15 M sucrose control	ln(OR)	confidence interval bounds: 95% lower	95% upper	Bonferroni lower	Bonferroni upper	p-value
byn-GAL4	w^{1118}	(pupae)	**88**	**95**	**99**	**109**	**0.0196**	–0.3977	0.4369	–0.5759	0.6151	**1.0000E+00**
			88	95	99	109						
	UAS-RNAi$^{dHHEX\text{-}V15721}$	(pupae)	**99**	**91**	**110**	**95**	**–0.0622**	–0.4772	0.3526	–0.6547	0.5296	**7.6324E–01**
			99	91	110	95						
MyoIA-GAL4	w^{1118}	(eclosers)	**1022**	**691**	**779**	**695**	**0.2772**	0.1341	0.4204	0.0712	0.4835	**1.2528E–04**
			343	229	309	275						
			351	259	193	156						
			328	203	277	264						
	UAS-RNAi$^{dHHEX\text{-}V15721}$	(eclosers)	**360**	**374**	**368**	**507**	**0.2821**	0.0799	0.4846	–0.0083	0.5732	**5.6701E–03**
			167	162	54	50						
			102	104	157	244						
			91	108	157	213						
esg-GAL4	w^{1118}	(eclosers)	**948**	**623**	**630**	**468**	**0.1225**	–0.0373	0.2822	–0.1075	0.3523	**1.2855E–01**
			326	187	257	150						
			289	221	160	133						
			333	215	213	185						
	UAS-RNAi$^{dHHEX\text{-}V15721}$	(eclosers)	**8**	**192**	**150**	**224**	**–2.7734**	–3.6572	–2.0346	–4.0945	–1.7726	**4.7473E–24**
			2	68	43	55						
			6	99	64	112						
			0	25	43	57						

Table S4. Driver survey, part 1. Knockdown of dHHEX in the tub$_P$ or esg domains results in sucrose intolerance. Data associated with Figure 3B: survival of progeny of various drivers crossed to UAS-RNAi^{dHHEX} or to w^{1118} (genetic background control) on 0.15 M and 1.0 M sucrose. Pupariation was scored for tub$_P$-GAL4, sns$_{GCN}$-GAL4, and byn-GAL4; eclosion was scored for Dilp2-GAL4, MyoIA-GAL4, and esg-GAL4. In the "scoring" columns, bold numbers are total counts, and counts for individual trials are listed immediately underneath them.

Table S5 – driver survey, part 2

(data associated with Figure 3C)

		scoring					confidence interval bounds				
		knockdown		background			95%		Bonferroni		
**Driver**	**Stage**	1.0 M sucrose	0.15 M sucrose	1.0 M sucrose	0.15 M sucrose	**ln(OR)**	lower	upper	lower	upper	**p-value**
Dot-GAL4	(pupae)	**164**	**162**	**169**	**179**	**0.0697**	–0.2439	0.3834	–0.3792	0.5190	**6.9997E–01**
		45	45	42	43						
		42	43	39	47						
		38	39	43	47						
		39	35	45	42						
	(eclosers)	**43**	**160**	**168**	**179**	**–1.2484**	–1.6723	–0.8381	–1.8603	–0.6684	**1.3922E–10**
		13	43	42	43						
		13	43	38	47						
		10	39	43	47						
		7	35	45	42						
Cg-GAL4	(pupae)	**219**	**227**	**259**	**266**	**–0.0092**	–0.2697	0.2513	–0.3829	0.3643	**9.4874E–01**
		38	40	44	44						
		41	33	40	46						
		34	46	44	43						
		34	33	44	42						
		37	37	44	45						
		35	38	43	46						
srp.Hemo-GAL4	(pupae)	**106**	**79**	**42**	**66**	**0.7434**	0.2341	1.2606	0.0215	1.4832	**2.5370E–03**
		31	15	11	21						
		27	31	20	21						
		48	33	11	24						
GMH5	(pupae)	**102**	**124**	**129**	**119**	**–0.2754**	–0.6539	0.1017	–0.8168	0.2628	**1.4185E–01**
		34	38	46	43						
		35	36	46	47						
		33	50	37	29						

Table S5. Driver survey, part 2. Knockdown of dHHEX in the Dot domain results in sucrose intolerance. Data associated with Figure 3C: survival of progeny of various drivers crossed to UAS-RNAi^{dHHEX} or to w^{1118} (genetic background control) on 0.15 M and 1.0 M sucrose. Pupariation (for 50 embryos per trial) was scored for Dot-GAL4, Cg-GAL4, srp.Hemo-GAL4, and GMH5. Eclosion was also scored for Dot-GAL4. In the "scoring" columns, bold numbers are total counts, and counts for individual trials are listed immediately underneath them.

Table S6 – circulating glucose
(data associated with Figure 4A)

hemolymph glucose (mg/dL)		
(control)		(experimental)
63.0	mean	71.1
10.3	SD	13.3
62.8	median	71.7
54.8-70.9	IQR	62.3-76.7
45.7-82.5	range	50.9-97.6
		97.6
		96.4
		91.6 93.0
		85.8
82.5		
		77.9
76.7		76.7
73.2 73.4 73.4		74.0
		71.7 71.7 72.1 72.9 73.0
67.4 68.6		
65.1 66.2		65.8 66.0
		63.9
61.6 62.8		61.6 62.3 62.5 62.8
59.3 60.2		59.0 60.4
55.4		
53.5 54.2		54.0
		52.9
50.6		50.9
45.7 46.9		

Table S6. Cg>RNAi$^{dHHEX\text{-}V15721}$, Dcr-2 larvae are hyperglycemic. Data associated with Figure 4A. We measured hemolymph glucose of groups of 5–8 wandering third-instar females reared on 1.0 M sucrose food.

Table S7 – body mass
(data associated with Figure 4B–C)

per-animal mass (mg)

(control)	**larvae**	(experimental)	(control)	**adults**	(experimental)
1.42	mean	1.32	1.00	mean	0.85
0.09	SD	0.05	0.09	SD	0.09
1.42	median	1.31	0.98	median	0.83
1.38-1.47	IQR	1.28-1.38	0.93-1.07	IQR	0.78-0.93
1.13-1.80	range	1.23-1.39	0.81-1.22	range	0.65-1.03
1.80					
1.54					
1.51 1.51 1.52 1.52 1.53					
1.48 1.48 1.48 1.49 1.50 1.50					
1.45 1.45 1.45 1.45 1.45 1.47 1.47 1.47 1.47					
1.43 1.43 1.43 1.43 1.43 1.43 1.44					
1.40 1.40 1.40 1.40 1.42 1.42 1.42 1.42 1.42		1.39			
1.36 1.38 1.38 1.38 1.38 1.38 1.38 1.39 1.39 1.39 1.39		1.38 1.38 1.38			
1.33 1.33 1.34 1.34		1.33 1.35			
1.30 1.30 1.30 1.30 1.32		1.30 1.31 1.32			
1.27 1.28		1.28 1.28 1.29			
1.26		1.27			
		1.23	1.22		
			1.19		
			1.16		
1.13			1.12 1.13 1.14		
			1.09 1.09 1.11		
			1.06 1.06 1.06 1.07 1.07 1.07 1.07 1.08 1.08		
			1.05 1.05		1.03
			1.00 1.00 1.01 1.01 1.02		1.00 1.01
			0.97 0.97 0.97 0.98 0.98 0.98 0.98 0.98		0.97 0.97 0.97 0.99 0.99
			0.94 0.94 0.95 0.96 0.96 0.96		0.95 0.95 0.95 0.95 0.95
			0.91 0.91 0.91 0.91 0.92 0.92 0.92 0.92 0.92 0.92 0.93 0.93		0.91 0.91 0.92 0.93 0.93
			0.89 0.90		0.88 0.88 0.89 0.89
					0.85 0.86 0.86
					0.83 0.83 0.83 0.83
			0.81 0.82		0.79 0.79 0.79 0.80 0.80 0.80 0.80 0.80 0.80 0.81 0.82
					0.76 0.77 0.77 0.77 0.78 0.78 0.78 0.78 0.78
					0.73
					0.70
					0.67 0.69
					0.65

Table S7. Cg>RNAi$^{dHHEX\text{-}V15721}$, Dcr-2 flies have reduced body mass. Data associated with Figure 4B–C. We measured mean per-animal mass of groups of 6–10 wandering third-instar and newly eclosed adult females reared on 1.0 M sucrose food.

Table S8 – triglycerides
(data associated with Figure 4D–E)

triglycerides (µg/mg tissue)					
(control)	**larvae**	(experimental)	(control)	**adults**	(experimental)
62.86	mean	51.11	53.24	mean	40.93
12.32	SD	11.47	14.53	SD	8.09
63.29	median	48.65	50.72	median	41.19
52.59-70.27	IQR	42.76-61.78	45.38-57.01	IQR	37.06-45.55
31.81-88.92	range	34.35-67.69	38.46-101.71	range	28.93-58.95
			101.71		
88.92					
81.31 81.93					
76.16 77.33 77.50 78.19					
74.01					
69.04 69.74 70.79					
64.74 64.78 65.85 66.80 66.95 67.40		64.55 65.93 67.69			
60.88 61.50 62.85 62.91 63.67			60.17		
59.06 59.44 59.68		59.00	56.14 56.97 57.06 58.35		58.95
53.96 55.06		55.20	52.81 53.39		
48.34 49.37 50.27 51.08 51.22		50.27	48.64		48.15 51.62 51.73
45.32 47.17 47.88		46.89 46.97 47.02	45.19 45.56 46.00 47.09		47.48
			40.33 43.96		40.91 41.46 41.50 42.47 42.99 43.61
		36.83 38.64	38.46		36.56 37.55 37.80 38.32 39.76
		34.35			
31.81					28.93 29.23 29.28 30.23

Table S8. Cg>RNAi$^{dHHEX\text{-}V15721}$, Dcr-2 flies have reduced triglyceride levels. Data associated with Figure 4D–E. We measured mean whole-animal triglyceride levels (per mg tissue) for groups of 6–10 wandering third-instar and newly eclosed adult females reared on 1.0 M sucrose food.
